# NCM811–Sulfide Electrolyte Interfacial Degradation Mechanisms and Regulation Strategies in All‐Solid‐State Lithium Battery

**DOI:** 10.1002/cssc.202501033

**Published:** 2025-10-13

**Authors:** Haoyu Feng, Guanghan Zhu, Ziming Wan, Feng Ryan Wang, Zhangxiang Hao, Junrun Feng

**Affiliations:** ^1^ School of Science School of Chip Industry Hubei University of Technology Wuhan Hubei 430068 China; ^2^ Materials and Catalysis Laboratory Department of Chemical Engineering University College London London WC1E 7JE UK

**Keywords:** all‐solid‐state lithium battery, cathode/solid‐state electrolyte interface, degradation mechanism, LiNi_0.8_Co_0.1_Mn_0.1_O_2_, sulfide solid‐state electrolyte

## Abstract

The all‐solid‐state lithium battery (ASSLB) with LiNi_0.8_Co_0.1_Mn_0.1_O_2_ (NCM811) cathode and sulfide solid‐state electrolyte (SSE) represents a transformative technology, offering enhanced safety and high energy density through the complete elimination of flammable liquid electrolyte and enabling the lithium metal anode. However, its commercialization is fundamentally limited by complex instabilities at the NCM811/sulfide SSE interface, which trigger coupled mechanical, chemical, and electrochemical degradation. The solid/solid interface creates complex dynamic feedback loops: mechanical stress from anisotropic volume changes accelerates interfacial chemical reactions; chemical degradation progressively alters electrochemical behavior; and continuous electrochemical cycling induces further mechanical instability. This multiscale coupling manifests as progressive contact loss, microcracks, detrimental space charge layer, and impedance growth, which collectively compromise performance under demanding conditions. This review establishes a coherent mechanistic framework to understand these highly interdependent degradation pathways, and systematically evaluates various stabilization strategies, including targeted surface modification, strategic bulk engineering, and innovative synergistic design approaches that specifically address the inherently coupled interface instability. Despite progress, intrinsic material incompatibilities persist, necessitating breakthroughs in materials design, interface engineering, characterization, and manufacturing. This work provides fundamental mechanistic insights into solid‐state electrochemistry and practical guidance for developing commercially viable ASSLB.

## Graphical abstract

This review focuses on the cathode/solid‐state electrolyte interfacial challenges in NCM811‐sulfide electrolyte systems, highlighting how the mechanical‐chemical‐electrochemical coupling failure limits performance. It evaluates stabilization strategies—including surface modification, bulk engineering, synergistic design, and electrolyte optimization—and proposes critical research directions for viable, high‐energy‐density all‐solid‐state lithium battery.

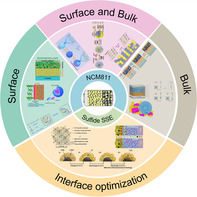

## 1 Introduction

The global energy transition demands advanced energy storage technologies beyond the current lithium‐ion battery (LIB).^[^ ^1^, ^2^, ^3^ ^]^ While LIB has revolutionized portable electronics and electric vehicles, its conventional flammable organic liquid electrolyte poses safety hazards and restricts the electrochemical window for high‐energy electrode materials.^[^ ^4^, ^5^, ^6^ ^]^ The all‐solid‐state lithium battery (ASSLB) replaces the liquid electrolyte with a solid‐state electrolyte (SSE), enhancing safety and enabling potentially higher energy density through compatibility with lithium metal anodes.^[^ ^7^, ^8^, ^9^, ^10^ ^]^ However, this architectural transformation introduces complex scientific challenges, particularly at the cathode/SSE interface, where mechanical, chemical, and electrochemical processes are intimately coupled.^[^ ^11^, ^12^, ^13^ ^]^ The success of ASSLB critically depends on developing SSE that can maintain interfacial stability under high‐voltage operation while providing the necessary ionic conductivity and mechanical properties.

Among various SSE candidates, sulfide SSE has emerged as particularly promising due to its unique combination of properties.^[^ ^14^, ^15^, ^16^, ^17^, ^18^ ^]^ Unlike traditional SSE systems such as polymers, oxides, and halides that struggle to simultaneously achieve high ionic conductivity, wide electrochemical stability, and favorable mechanical properties, sulfide SSE exhibits room‐temperature ionic conductivity (10^−3^‐10^−2^ S cm^−1^), comparable to that of liquid electrolyte, and intrinsic deformability that facilitates intimate solid‐solid contact without requiring high‐temperature sintering.^[^ ^19^, ^20^, ^21^, ^22^, ^23^, ^24^ ^]^ However, understanding and controlling the complex interfacial phenomena between sulfide SSE and high‐voltage cathode materials represents a fundamental materials science challenge, requiring new theoretical frameworks and experimental approaches.

**Figure 1:**
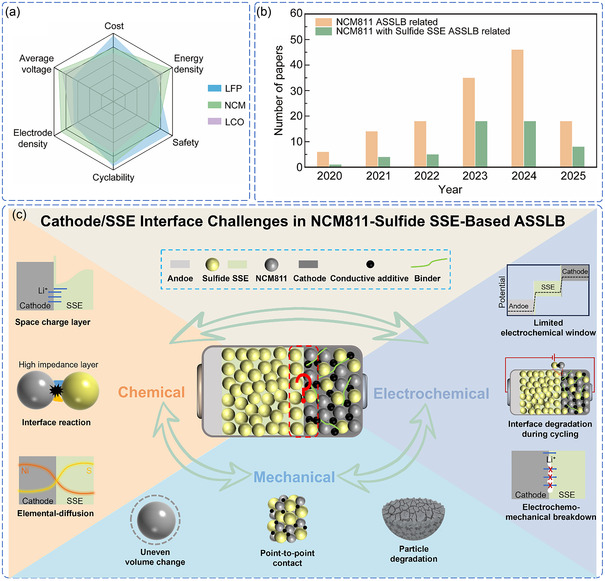
a) Comparative performance metrics of cathodes in ASSLB (LCO: LiCoO_2_, NCM: LiNi_0.8_Co_0.1_Mn_0.1_O_2_, LFP: LiFePO_4_). b) The number of publications about NCM811‐based ASSLB and NCM811‐sulfide SSE‐based ASSLB from 2020 to 2025 (keywords search from Web of Science, accessed April 15, 2025). c) Schematic illustration of multi‐mechanism coupling challenges at the cathode/SSE interface of the NCM811‐sulfide SSE‐based ASSLB.

Building on the promising properties of sulfide SSE, the selection of compatible cathode materials becomes crucial for realizing high‐performance ASSLB.^[^ ^25^, ^26^ ^]^ Among various candidates, LiNi_0.8_Co_0.1_Mn_0.1_O_2_ (NCM811) has emerged as particularly compelling due to its superior specific capacity (>200 mAh g^−1^) and high operating voltage (>4.3 V vs Li^+^/Li), significantly exceeding traditional cathode materials like LiCoO_2_ and LiFePO_4_.^[^ ^27^, ^28^, ^29^, ^30^, ^31^ ^]^ This performance advantage is illustrated in **Figure** **1**a. However, this high‐voltage operation creates extreme conditions at the cathode/SSE interface, where mechanical stress from volume changes, chemical reactions between dissimilar materials, and electrochemical instabilities converge to create unprecedented challenges.^[^ ^32^ ^]^ The combination of NCM811's aggressive electrochemical environment and sulfide SSE's unique chemical reactivity makes this interface a critical bottleneck for ASSLB development.

The scientific and technological significance of NCM811‐sulfide SSE‐based ASSLB has attracted intense interest from both academia and industry. The exponential growth in research publications since 2020, as illustrated in Figure 1b, reflects the urgent need to address fundamental interface challenges in these systems. The practical importance of addressing these interfacial challenges is underscored by significant industrial investments and milestones. Toyota has progressed from battery prototypes (2019) to vehicle testing (2020) and plans mass production by 2027–2028, while LG Chem and UC San Diego demonstrated a μSi/Li_6_PS_5_Cl/NCM811 cell retaining 80% capacity after 500 cycles.^[^ ^33^, ^34^ ^]^ Despite these advances, the fundamental complexity of the cathode/SSE interface, spanning atomic‐scale reactions to microscale mechanical evolution, demands new theoretical frameworks that can bridge laboratory discoveries with industrial requirements.

Recent investigations have revealed that interface degradation in NCM811‐sulfide SSE systems involves intricate coupling between three primary mechanisms, as systematically illustrated in Figure 1c.^[^ ^35^, ^36^, ^37^, ^38^ ^]^ Mechanical degradation arises from interfacial contact loss and heterogeneous volume changes during cycling. Chemical degradation proceeds through interfacial reactions and elemental interdiffusion that alter local composition and properties. Electrochemical degradation manifests as charge transfer impedance and ion transport limitations that evolve with cycling. Unlike conventional LIB, where these processes can be treated independently, the solid–solid nature of ASSLB interfaces creates dynamic feedback loops where mechanical stress accelerates chemical reactions, chemical changes modify electrochemical behavior, and electrochemical cycling induces further mechanical instability. While previous reviews have addressed various aspects of nickel‐rich cathode and sulfide SSE, they have primarily focused on material classifications and phenomenological observations, leaving the fundamental mechanisms governing cathode/SSE interface evolution poorly understood.^[^ ^39^, ^40^, ^41^, ^42^ ^]^ The complex coupling between mechanical, chemical, and electrochemical processes at the NCM811/sulfide SSE interface requires new theoretical frameworks that can elucidate these interdependencies and predict interface behavior under high‐voltage operation. Such a mechanistic understanding is essential for developing rational design strategies that can stabilize these critical interfaces. The systematic investigation of NCM811/sulfide SSE interface science thus addresses both immediate technological needs and fundamental scientific questions, potentially establishing new design principles for next‐generation solid‐state energy storage systems.

This review establishes a comprehensive framework for understanding and controlling interface challenges in NCM811‐sulfide SSE systems. We systematize the cross‐scale coupling mechanisms of mechanical, chemical, and electrochemical degradation, revealing their dynamic interactions and feedback loops. Subsequently, we evaluate interface stabilization strategies, including surface modification, bulk engineering, and synergistic design approaches, to analyze how different interventions affect the coupled degradation processes. Finally, we identify critical research directions encompassing molecular design principles, advanced characterization techniques, structure‐performance relationships, standardized protocols, and manufacturing scalability. These insights provide both a fundamental understanding of solid‐state electrochemistry and practical guidelines for realizing stable, high‐performance ASSLB.

## 2 Cathode/SSE Interface Challenges in NCM811‐Sulfide SSE‐Based ASSLB

The stability of the cathode/SSE interface is the core challenge limiting the development of high‐energy‐density NCM811‐sulfide SSE‐based ASSLB. The failure mechanism arises from the destructive‐coupling of mechanical degradation, chemical incompatibility, and electrochemical instability. This coupling exhibits significant multiscale features: atomic‐level interfacial chemical reactions can trigger microscale mechanical damage, while the high operating voltage induces interfacial side reactions that propagate structural degradation into the bulk phase. In turn, mechanical stress concentration exposes fresh surfaces and increases the active reaction area, while the increase in electrochemical impedance intensifies local potential gradients, forming a dynamically reinforced feedback loop. These processes collectively govern the long‐term stability of the interface. Therefore, understanding the “mechanical–chemical–electrochemical” coupling mechanism is crucial for achieving high‐performance ASSLB.

### 2.1 Mechanical Contact Degradation and Evolution

**Figure 2:**
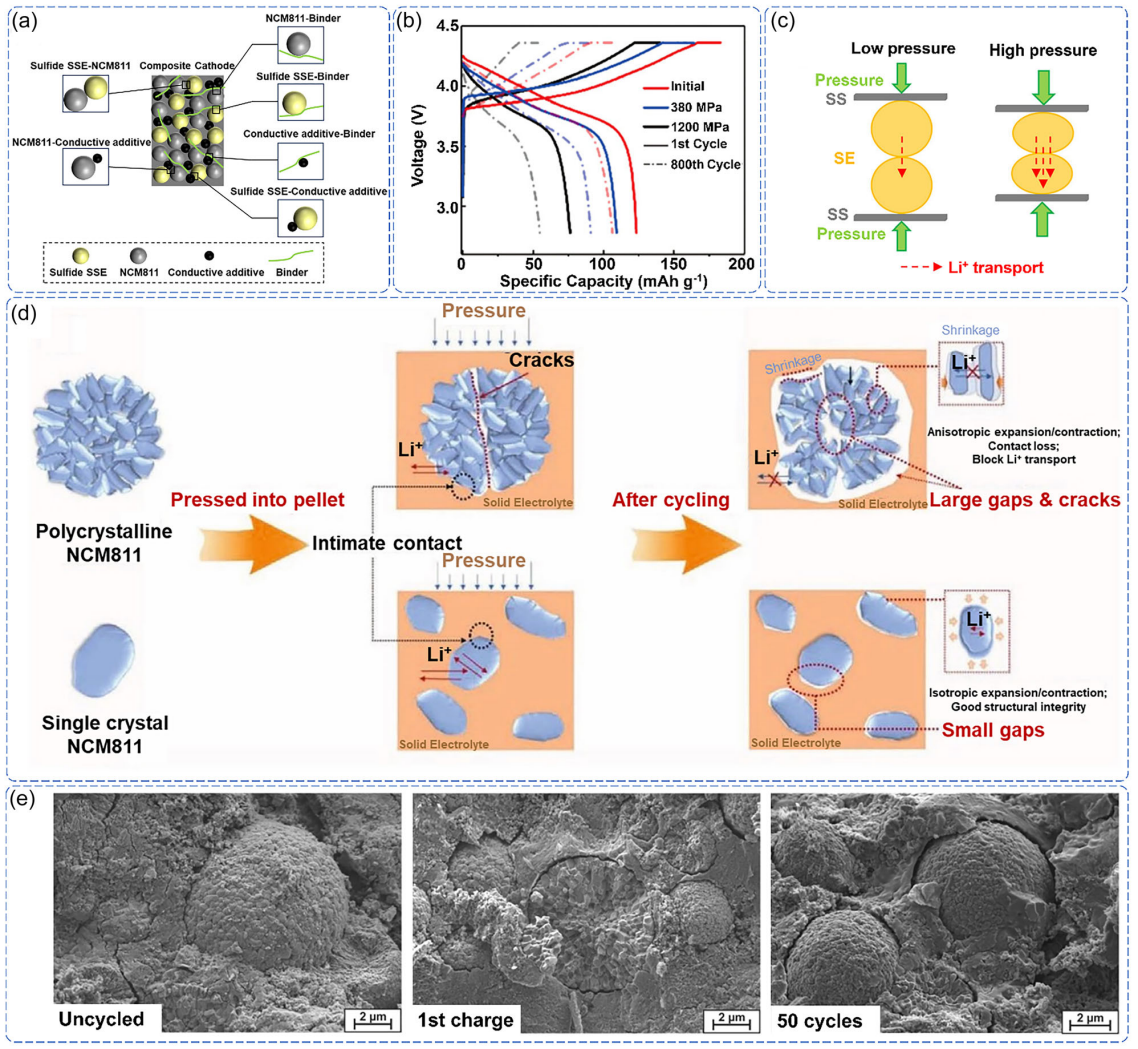
a) Schematic illustration of various interfaces within the NCM811 composite cathode in ASSLB. b) ASSLB cycling performance under different fabrication pressures. Reproduced with permission.^[^ ^47^ ^]^ Copyright 2023, Royal Society of Chemistry. c) Effect of pressure on Li^+^ transport in SSE. Reproduced with permission.^[^ ^50^ ^]^ Copyright 2023, Wiley‐VCH. d) Schematic illustration of cross‐sectional morphological changes in single‐crystal and polycrystalline NCM811 composite cathodes during electrode compaction. Reproduced with permission.^[^ ^57^ ^]^ Copyright 2020, Wiley‐VCH. e) Scanning electron microscopy (SEM) images of NCM811‐β‐Li_3_PS_4_ composite cathode before cycling (left), after first charge (middle), and after 50 cycles (right). Reproduced with permission.^[^ ^61^ ^]^ Copyright 2017, American Chemical Society.

Unlike liquid electrolyte systems, where molecular‐level contact is naturally achieved through wetting, the solid‐state battery faces fundamental solid–solid interface challenges where discrete particle surfaces with inherent roughness and geometric irregularities cannot spontaneously conform to achieve intimate contact. In NCM811‐sulfide SSE‐based ASSLB, this solid–solid contact limitation manifests through two critical types of mechanical interfaces: the primary cathode/SSE interface, where NCM811‐based electrodes directly contact sulfide SSE, and secondary inter‐particle interfaces within the composite cathode, including NCM811/conductive additives, sulfide SSE/binders, and conductive additives/binders (**Figure** **2**a).^[^ ^12^, ^43^ ^]^

The cathode/SSE interface represents the most mechanically incompatible contact point, where the rigid nature of both materials and their surface irregularities create inevitable microscopic gaps and voids, fundamentally hindering ionic and electronic transport.^[^ ^44^ ^]^ Additionally, the poor solid–solid interfacial contact between NCM811 and sulfide SSE prevents spontaneous adhesion, requiring external force to establish contact.^[^ ^45^, ^46^ ^]^ The necessity for appropriate fabrication pressure to achieve basic interfacial contact reveals the profound mechanical mismatch between these materials, while the material‐specific pressure requirements expose even deeper incompatibilities.^[^ ^47^, ^48^, ^49^ ^]^ This incompatibility manifests in multiple ways. Zhang et al. demonstrated that adequate densification requires high pressures (>380 MPa) that undermine‐mechanical integrity, with crack formation occurring even under controlled conditions, leading to a significant reduction in the initial capacity of the ASSLB (Figure 2b).^[^ ^47^ ^]^ As fabrication pressure increases, the SSE surface becomes smoother, and porosity decreases, resulting in significantly increased density and ionic conductivity (Figure 2c).^[^ ^50^ ^]^

Moreover, different sulfide SSE types exhibit drastically different pressure requirements: Li_6_PS_5_Br needs 1000–1500 MPa for ionic conductivity enhancement but suffers irreversible strain at ≥500 MPa, while Li_3_PS_4_ fails at only ≈100 MPa.^[^ ^51^, ^52^ ^]^ This behavior varies even within the same composition: crystalline β‐Li_3_PS_4_ undergoes irreversible deformation while glassy g‐Li_3_PS_4_ shows reversible elastic changes, highlighting the unpredictable nature of these interfaces.^[^ ^52^, ^53^ ^]^ These material‐specific limitations represent significant engineering challenges that require innovative approaches to address. Beyond the primary interface challenges, secondary inter‐particle interfaces introduce additional mechanical complexity that exacerbates these fundamental problems. These interfaces create a mechanically heterogeneous network where large differences in elastic moduli (soft binders < sulfide SSE < hard NCM811) make stress concentration unavoidable.^[^ ^54^ ^]^ The point‐contact geometry of conductive additives creates stress singularities that exceed material fracture strength under minimal loading, while the multi‐component nature ensures that any single component failure compromises the entire electron transport network. Critically, sulfide SSE must simultaneously accommodate stress from multiple interface types, creating competing mechanical demands that establish the foundation for cascade failure mechanisms.

During battery operation, electrochemically induced volume changes exacerbate these structural problems. NCM811's anisotropic expansion conflicts with sulfide SSE's isotropic response, creating stress states that are difficult to accommodate. Even “optimal” stacking pressure tends to delay rather than prevent interfacial degradation, suggesting that the problem is primarily thermodynamic rather than kinetic. Studies in conventional liquid electrolyte systems demonstrate that single‐crystal NCM811 exhibits reduced volume change (2.4%) compared to polycrystalline variants (5.0%) during cycling. However, these volume changes, already problematic in liquid systems, are expected to be amplified in rigid solid‐state configurations where mechanical accommodation is further limited.^[^ ^55^, ^56^ ^]^ The work of Liu et al. demonstrates that SC‐NCM811's “superior” performance reveals reduced but persistent degradation, not true stability (Figure 2d).^[^ ^57^ ^]^ The H2 → H3 phase transition above 4.2 V introduces additional anisotropic strain, while limited sulfide SSE fracture toughness (e.g., K_IC_ ≈ 0.23 MPa·m^1/2^ for Li_6_PS_5_Cl) ensures that minor stress concentrations exceed critical thresholds.^[^ ^58^, ^59^, ^60^ ^]^ Further systematic studies on NCM811 volume evolution in solid‐state environments are critically needed.

The combination of structural vulnerability and cycling‐induced stress creates an unavoidable degradation pathway where mechanical contact failure represents a thermodynamically driven process rather than an engineering challenge. The cascade mechanism is inherent to the multi‐interface architecture: the mechanical property hierarchy ensures that secondary interface degradation precedes primary interface failure. Hwang et al. and Koerver et al. have demonstrated that this sequential failure creates current localization at the NCM811/sulfide SSE interface, establishing positive feedback loops where mechanical stress and electrochemical activity mutually amplify degradation (Figure 2e).^[^ ^61^, ^62^ ^]^ Volume change incompatibility ensures that some interfaces will always fail under cycling conditions, regardless of optimization efforts. This mechanical degradation blocks ionic transport and generates high electrochemical potential gradients that expose fresh, reactive surfaces, establishing the critical link to chemical degradation mechanisms discussed in the following section.

### 2.2 Chemical Interface Degradation

The mechanical contact failures described above expose fresh, highly reactive surfaces at the cathode/SSE interface, creating active sites for thermodynamically driven chemical degradation. While mechanical failure initiates the process, subsequent interfacial chemical reactions amplify and perpetuate degradation through irreversible interphase formation, progressively increasing interfacial resistance. This chemical degradation represents a secondary but critical failure mode that transforms localized mechanical damage into system‐wide performance deterioration. Understanding this amplification mechanism requires analyzing both the thermodynamic driving forces that make reactions spontaneous and the kinetic barriers that govern‐reaction rates and pathways.

**Figure 3:**
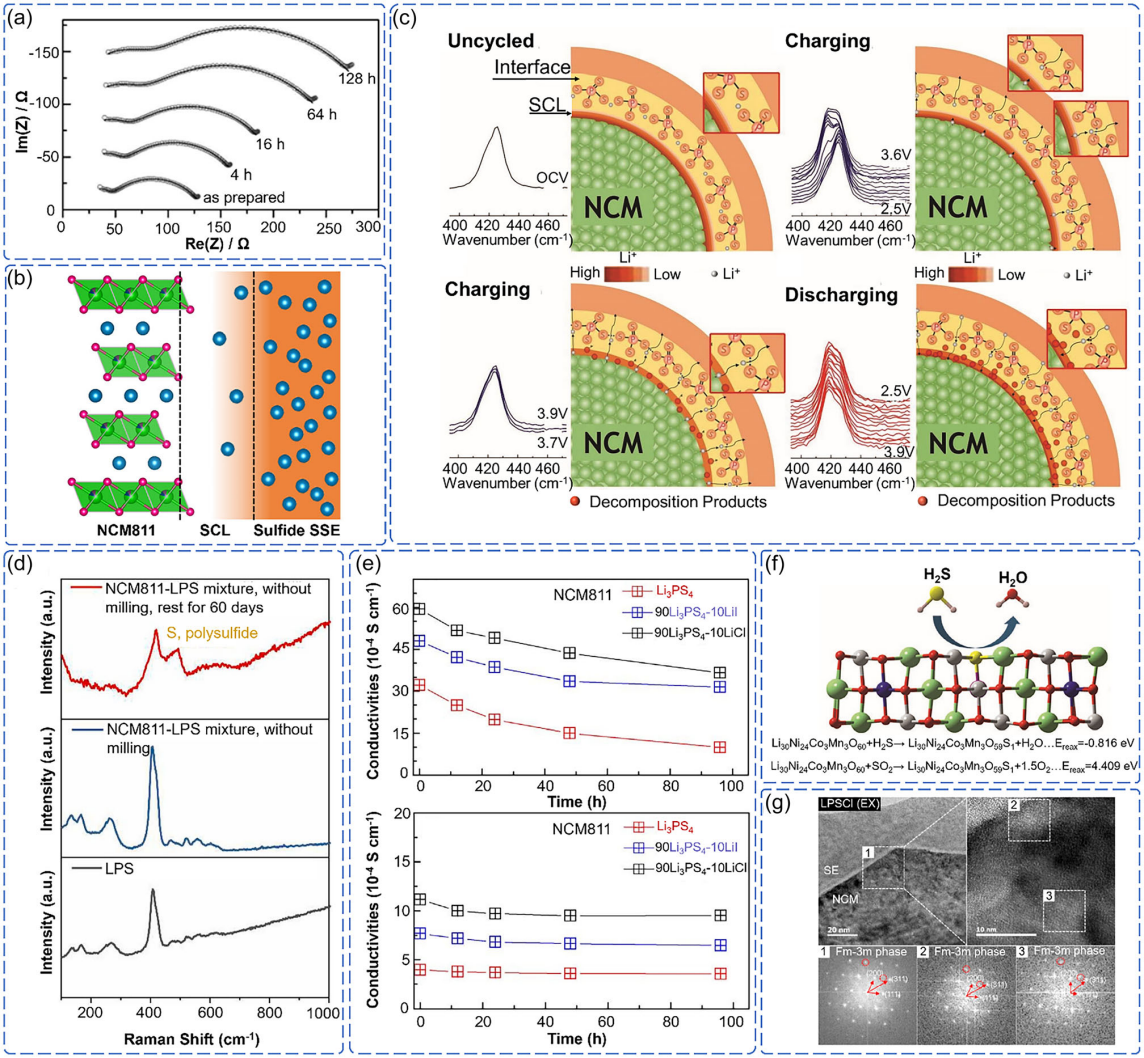
a) Time‐dependent evolution of the electrochemical impedance spectroscopy and fit for a mixture of NCM811 and β‐Li_3_PS_4_ without any applications of current or potential in a house‐made press cell over time. Reproduced with permission.^[^ ^61^ ^]^ Copyright 2017, American Chemical Society. b) SCL formation driven by Li^+^ depletion at the NCM811/sulfide SSE interface. Reproduced with permission.^[^ ^66^ ^]^ Copyright 2023, Springer Nature. c) In situ Raman‐based schematic of interfacial evolution during cycling, correlating P‐S bond vibration states (PS_4_^3−^ group) at NCM811/Li_6_PS_5_Cl interface. Reproduced with permission.^[^ ^69^ ^]^ Copyright 2019, Wiley‐VCH. d) Raman spectra of Li_7_P_3_S_11_, non‐milled mixture of NCM811/Li_7_P_3_S_11_, non‐milled mixture of NCM811 and Li_7_P_3_S_11_ that has been rest for 60 days. Reproduced with permission.^[^ ^70^ ^]^ Copyright 2023, Elsevier. e) Ionic conductivities of NCM811/sulfide SSE mixtures: (top) without preheating and (bottom) after preheating at 80 °C. Reproduced with permission.^[^ ^71^ ^]^ Copyright 2018, Elsevier. f) Reaction mechanism schematic of H_2_S‐induced surface degradation on NCM811 cathode. Reproduced with permission.^[^ ^74^ ^]^ Copyright 2023, Wiley‐VCH. g) Post‐cycling high‐resolution transmission electron microscopy image and corresponding Fast Fourier Transform patterns of NCM811/Li_6_PS_5_Cl interface in ASSLB. Reproduced with permission.^[^ ^75^ ^]^ Copyright 2023, American Chemical Society.

The mechanically compromised interfaces provide ideal conditions for thermodynamically driven chemical reactions. The exposed fresh surfaces eliminate kinetic barriers that normally protect buried interfaces, while the inherent redox incompatibility between highly oxidizing NCM811 (4.0–4.3 V vs Li^+^/Li) and the strongly reducing sulfide SSE creates a powerful driving force for spontaneous charge transfer.^[^ ^63^, ^64^ ^]^ This redox mismatch results in interfacial reactions that generate insulating by‐products, including metal sulfides, phosphides, and oxidized derivatives, progressively increasing interfacial resistance.^[^ ^65^, ^66^, ^67^, ^68^ ^]^ However, reaction kinetics rather than thermodynamics ultimately control degradation rates. Koerver et al. have demonstrated this through impedance measurements which showed negligible initial degradation despite strong thermodynamic driving forces, with substantial deterioration emerging only after 16 h (**Figure** **3**a).^[^ ^61^ ^]^ This time‐dependent behavior confirms that interfacial chemical degradation proceeds as a kinetically controlled, multi‐step process where activation barriers determine reaction pathways and rates.

In addition to direct chemical reactions, the formation of a space charge layer (SCL) at mechanically compromised interfaces creates additional degradation pathways. When fresh NCM811 surfaces contact the sulfide SSE, chemical potential gradients drive Li^+^ migration until electrochemical equilibrium is established, creating Li^+^‐depleted regions at the interface (Figure 3b).^[^ ^66^ ^]^ This charge redistribution fundamentally alters the local chemical environment and reaction kinetics. Zhang et al. demonstrated through in situ electrochemical impedance spectroscopy that Li^+^ redistribution during initial charging significantly elevates diffusion barriers and interfacial impedance.^[^ ^69^ ^]^ More critically, in situ Raman spectroscopy revealed that Li^+^ migration triggers dynamic restructuring of PS_4_^3−^ groups at the interface, indicating that SCL effects extend beyond simple charge redistribution to drive fundamental changes in local bonding and reactivity (Figure 3c).^[^ ^69^ ^]^ These SCL‐induced chemical environment changes create new reaction pathways that would not exist at pristine interfaces.

The coupling between mechanical failure and chemical degradation creates synergistic effects that exceed the sum of individual contributions. Mechanical stress concentrates at chemically weakened interfaces, while chemical reactions preferentially occur at mechanically damaged sites where activation barriers are reduced. This mechanical–chemical coupling explains why degradation rates accelerate over time rather than remaining constant. Liang et al. demonstrated this synergy by showing that mechanical energy during processing dramatically accelerates chemical reaction kinetics, while chemical degradation products create stress concentrations that promote further mechanical failure (Figure 3d).^[^ ^70^ ^]^ Environmental conditions further amplify these synergistic degradation mechanisms, transforming controlled interfacial reactions into uncontrolled cascade failures. The extreme air sensitivity of sulfide SSE becomes particularly problematic when mechanical failure exposes fresh, unprotected surfaces. Chen et al. demonstrated that while controlled heating (80 °C) can form protective passivation layers, room temperature conditions allow persistent degradation that continuously compromises conductivity (Figure 3e).^[^ ^71^ ^]^ Moisture‐induced H_2_S evolution creates additional reaction pathways that simultaneously degrade both SSE structure and NCM811 surface chemistry. H_2_S release rates escalate dramatically with increasing temperature and relative humidity.^[^ ^72^, ^73^ ^]^ Choi et al. and Jung et al. revealed that H_2_S‐mediated reduction transforms electrochemically active layered NCM811 (R‐3m) into inactive rock salt phases (Fm‐3m) while consuming active lithium sources and causing irreversible capacity loss (Figure 3f,g).^[^ ^74^, ^75^ ^]^ These environmental factors do not simply add to existing degradation—they fundamentally alter the reaction landscape by creating new failure modes and accelerating existing ones, demonstrating how external conditions can overwhelm even well‐designed interface protection strategies. To address these challenges, effective strategies to enhance air stability primarily include in situ H_2_S absorbers, bulk‐phase element substitution, and surface molecular engineering.^[^ ^73^, ^76^, ^77^ ^]^

The chemical degradation mechanisms described above create a self‐reinforcing failure cascade that fundamentally distinguishes solid‐state systems from conventional LIB. Mechanically exposed interfaces provide reactive sites for thermodynamically driven redox reactions, while SCL formation alters local chemical environments to create new degradation pathways inaccessible at pristine interfaces. The resulting insulating degradation products not only increase interfacial resistance but also generate volumetric stress that propagates mechanical cracks, exposing additional reactive surface area. This mechanical–chemical coupling establishes positive feedback loops where each type of degradation accelerates the other: higher interfacial impedance concentrates current density and elevates local potentials, reducing activation barriers for further chemical reactions, while chemical degradation products create stress concentrations that promote additional mechanical failure. Unlike reversible electrochemical processes, these degradation mechanisms are fundamentally irreversible and cumulative, ensuring that initial damage tends to propagate throughout the interface unless effectively mitigated. This irreversible nature transforms localized interface problems into system‐wide performance degradation, establishing chemical instability as the critical link between mechanical failure and the electrochemical degradation processes discussed in the following section.

### 2.3 Electrochemical Interface Degradation

The mechanical and chemical interface damage described above creates conditions for severe electrochemical instability, which represents the final stage of interface degradation. While pristine interfaces can temporarily withstand NCM811's high operating voltages (>4.3 V vs Li^+^/Li) through kinetic protection, compromised interfaces eliminate these barriers and expose reactive sites directly to electrochemical stress. This electrochemical degradation amplifies existing failure modes through irreversible feedback loops that drive system‐wide catastrophic failure. Understanding this process requires examining how electrochemical forces exploit interface vulnerabilities to trigger cascading failures.

Electrochemical degradation at compromised interfaces is thermodynamically driven by the voltage incompatibility between NCM811 and sulfide SSE. The Gibbs free energy change (Equation 1) and lithium chemical potential (Equation 2)^[^ ^78^, ^79^ ^]^

(1) $$\begin{array}{c} \Delta G=-nFE \end{array}$$

where *G* is the Gibbs free energy; *n* is the number of transferred electrons; *F* is the Faraday constant; and *E* is voltage.

(2) $$\begin{array}{c} \mu_{\text{Li}}=\mu_{\text{Li}}^{0}-eV \end{array}$$

where $\mu_{\text{Li}}^{0}$ is the chemical potential of Li metal, *e* is the elementary charge, and *V* is voltage.

Both equations indicate that higher operating voltages create stronger driving forces for interfacial reactions. At damaged interfaces where protective barriers have been eliminated, these thermodynamic forces can overcome kinetic limitations that normally stabilize pristine cathode/SSE contacts. Consequently, sulfide SSE experiences accelerated oxidative decomposition even at moderate voltages, with degradation rates dramatically increasing above 4.3 V vs Li^+^/Li, where both thermodynamic driving forces and kinetic accessibility combine to drive rapid structural breakdown.

**Figure 4:**
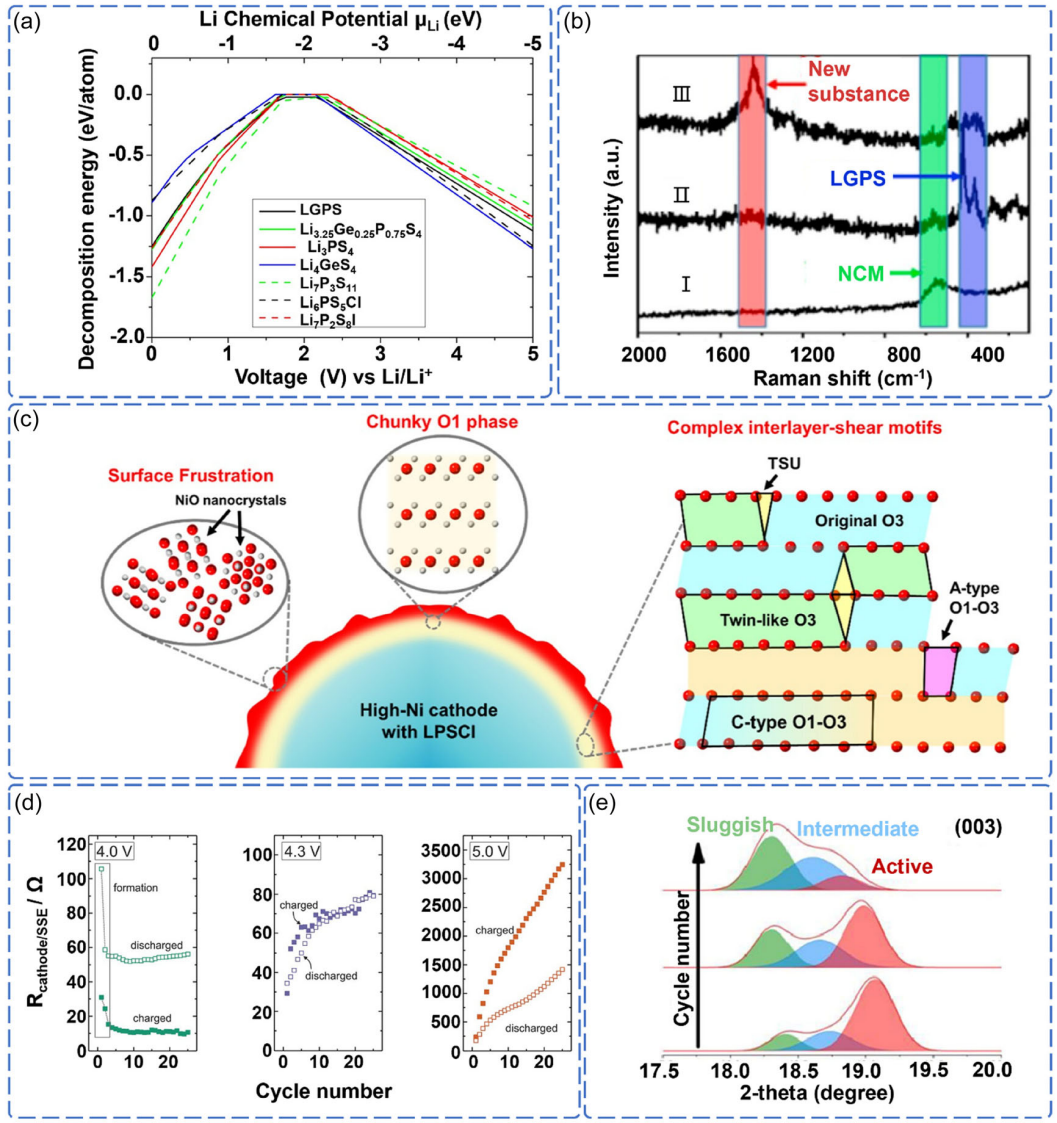
a) Decomposition energy of sulfide SSE as a function of the applied voltage or Li chemical potential μ_Li_. Reproduced with permission.^[^ ^80^ ^]^ Copyright 2015, American Chemical Society. b) Raman spectra of NCM811, Li_10_GeP_2_S_12_, and NCM811/Li_10_GeP_2_S_12_ interface from NCM811 cathode layer after cycling. Reproduced with permission.^[^ ^82^ ^]^ Copyright 2020, Elsevier. c) Electrochemically triggered atomic‐scale degradation of NCM811 cathode in ASSLB. Reproduced with permission.^[^ ^83^ ^]^ Copyright 2024, American Chemical Society. d) Evolution of R_cathode/SSE_ upon cycling for Li–In/β‐Li_3_PS_4_/NCM811+β‐Li_3_PS_4_ batteries charged to different upper cutoff voltages between 4.0 and 5.0 V. Reproduced with permission.^[^ ^85^ ^]^ Copyright 2017, Royal Society of Chemistry. e) Fitted peaks of X‐ray diffraction (XRD) spectra of NCM811 (003) at different cycle numbers. Reproduced with permission.^[^ ^86^ ^]^ Copyright 2023, Elsevier.

While thermodynamic analysis predicts rapid degradation, the actual process is controlled by complex kinetic factors that depend critically on interface conditions. This kinetic control explains a fundamental paradox. First‐principles calculations predict sulfide SSE oxidation at low voltages (2.0–2.3 V vs Li^+^/Li) (**Figure** **4**a).^[^ ^80^ ^]^ Specifically, the electrochemical stability windows of Li_10_GeP_2_S_12_ and Li_6_PS_5_Cl are 1.71–2.14 V vs Li^+^/Li and 1.71–2.01 V vs Li^+^/Li, respectively.^[^ ^80^, ^81^ ^]^ However, experiments show that sulfide SSE remains relatively stable up to 5 V vs Li^+^/Li, which contrasts with the computational predictions. At pristine interfaces, slow reaction kinetics and passivating layer formation provide temporary protection despite strong thermodynamic driving forces. However, this kinetic protection is eliminated at mechanically and chemically damaged interfaces where fresh, reactive surfaces are directly exposed to electrochemical stress. Consequently, the same sulfide SSE appears stable in pristine cells undergoes rapid degradation when interface damage removes kinetic barriers, transforming thermodynamically possible reactions into kinetically accessible pathways.

At damaged interfaces exposed to high voltages, specific electrochemical mechanisms drive rapid degradation through oxygen loss and phase transformation. Li et al. demonstrated that highly reactive Ni^4+^ species on charged NCM811 surfaces directly trigger irreversible reactions with sulfide SSE, generating insulating degradation products at the interface (Figure 4b).^[^ ^82^ ^]^ This electrochemical attack simultaneously induces structural collapse within NCM811 itself. Wang et al. revealed through atomic‐scale analysis that oxygen loss drives misoriented rock‐salt phase transformation, creating complex shear patterns that cause severe mechanical damage to the cathode structure (Figure 4c).^[^ ^83^ ^]^ This electrochemical‐mechanical coupling establishes a destructive feedback loop where electrochemical reactions weaken mechanical integrity, while mechanical damage exposes additional reactive surfaces to electrochemical attack.

The severity of electrochemical degradation exhibits a sharp voltage dependence, with critical thresholds that determine whether damage remains localized or propagates throughout the cathode. Above 4.3 V versus Li^+^/Li, experiments confirm that decomposition at the NCM811/β‐Li_3_PS_4_ interface leads to the formation of ‐S‐S‐ bonds and P–O_*x*_ species, while simultaneously triggering redox‐active layer formation that extends degradation beyond the immediate interface (Figure 4d).^[^ ^84^, ^85^ ^]^ More critically, this electrochemical attack propagates into the NCM811 bulk through lattice strain mechanisms. Liu et al. demonstrated that intense interfacial reactions during initial charging create structurally compromised regions within NCM811 crystals that progressively expand with continued cycling, ultimately leading to bulk structural collapse (Figure 4e).^[^ ^86^ ^]^ This interface‐to‐bulk propagation transforms localized electrochemical damage into system‐wide cathode degradation, establishing electrochemical instability as a mechanism that can compromise entire electrode structures rather than just surface regions.

Electrochemical degradation represents the most destructive stage of interface failure because it creates irreversible, system‐wide damage that far exceeds the localized effects of mechanical or chemical degradation alone. Unlike the gradual progression of mechanical contact loss or chemical interphase formation, electrochemical instability triggers rapid bulk structural collapse that propagates throughout the cathode material. This creates multiple self‐reinforcing feedback loops: electrochemical reactions generate degradation products that increase interfacial impedance, leading to current localization and higher local potentials that accelerate further reactions; simultaneously, structural damage from phase transformations creates mechanical stress that propagates cracks and exposes additional reactive surfaces to electrochemical attack. The cumulative effect transforms the three individual degradation modes—mechanical, chemical, and electrochemical—into a unified failure cascade where each mode amplifies the others, ensuring that any initial interface damage inevitably leads to complete system failure. This fundamental interconnectedness of failure modes establishes why conventional mitigation strategies that address individual degradation mechanisms cannot achieve long‐term stability in NCM811‐sulfide SSE systems.

## 3 Interface Modification Strategies between NCM811 and Sulfide SSE

**Figure 5:**
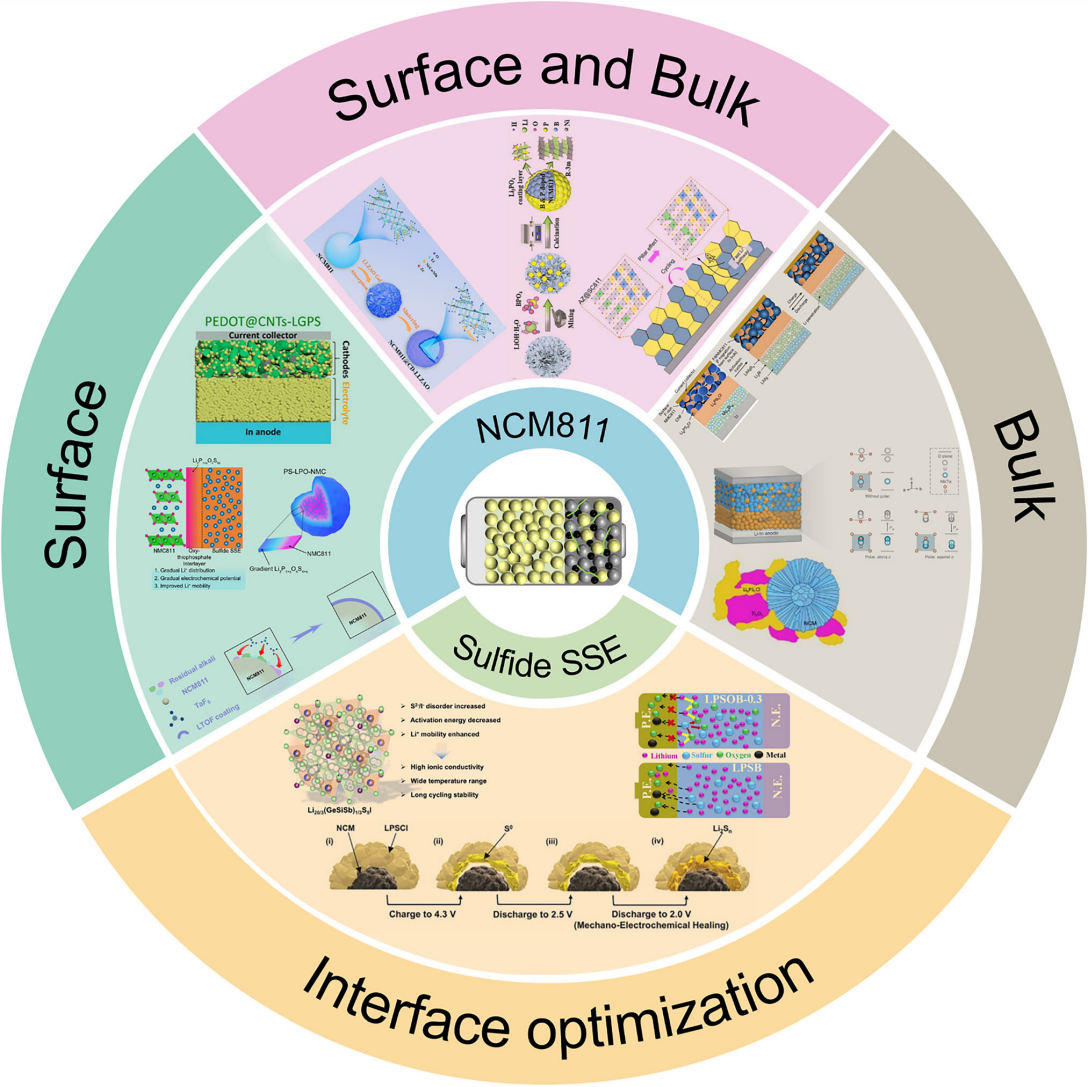
Schematic illustration of interface modification strategies between NCM811 and sulfide SSE. Reproduced with permission.^[^ ^94^ ^]^ Copyright 2020, American Chemical Society. Reproduced with permission.^[^ ^66^ ^]^ Copyright 2023, Springer Nature. Reproduced with permission.^[^ ^95^ ^]^ Copyright 2024, Wiley‐VCH. Reproduced with permission.^[^ ^97^ ^]^ Copyright 2023, Springer Nature. Reproduced with permission.^[^ ^98^ ^]^ Copyright 2024, American Chemical Society. Reproduced with permission.^[^ ^99^ ^]^ Copyright 2023, The Authors. Elsevier. Reproduced with permission.^[^ ^102^ ^]^ Copyright 2024, Elsevier. Reproduced with permission.^[^ ^103^ ^]^ Copyright 2023, Wiley‐VCH. Reproduced with permission.^[^ ^105^ ^]^ Copyright 2023, American Chemical Society. Reproduced with permission.^[^ ^77^ ^]^ Copyright 2019, Elsevier. Reproduced with permission.^[^ ^107^ ^]^ Copyright 2025, American Chemical Society. Reproduced with permission.^[^ ^84^ ^]^ Copyright 2025, Wiley‐VCH.

While the analysis in Section 2 reveals that interfacial instability between NCM811 and sulfide SSE poses significant and complex challenges involving mechanical–chemical–electrochemical coupling, extensive research efforts have developed various engineering strategies to address these problems, including surface modification of NCM811, bulk‐phase doping engineering, surface‐bulk phase synergistic regulation, and interfacial engineering of sulfide SSE (**Figure** **5**). These approaches have achieved notable improvements in laboratory settings, though they still face substantial challenges in addressing the multifaceted nature of interface degradation. This section systematically examines these strategies, analyzing their mechanisms, achievements, and remaining limitations to provide insights into future research directions for NCM811‐sulfide SSE systems.

### 3.1 Surface Modification Strategies of NCM811

Surface coating technology, as the most direct method of interfacial regulation, has received widespread attention in recent years (Table S1, Supporting Information). While early coating strategies primarily focused on traditional lithium‐ion conductors (such as LiAlO_2_,^[^ ^48^ ^]^ LiNbO_3_,^[^ ^57^, ^82^ ^]^ Li_2_O,^[^ ^87^ ^]^ LiOH,^[^ ^88^ ^]^ Li_3_PO_4_,^[^ ^89^ ^]^ Li_2_SiO_*x*_,^[^ ^90^ ^]^ Li_2_ZrO_3_,^[^ ^91^ ^]^ Li_3_VO_4_,^[^ ^92^ ^]^ Li_5_FeO_4_,^[^ ^93^ ^]^ etc.) to suppress direct (electro)chemical reactions at the interface, recent studies suggest that achieving true interfacial stability requires synergistic control of physical contact, chemical reactions, and charge transfer across multiple scales. Ideal coating materials should possess the following four key characteristics: 1) good mechanical flexibility, enabling strain accommodation and maintaining tight contact; 2) high chemical stability against both NCM811 and sulfide SSE under high‐voltage operation; 3) low electronic conductivity and high Li^+^ mobility, while suppressing harmful SCL formation; and 4) excellent electrochemical durability.

**Figure 6:**
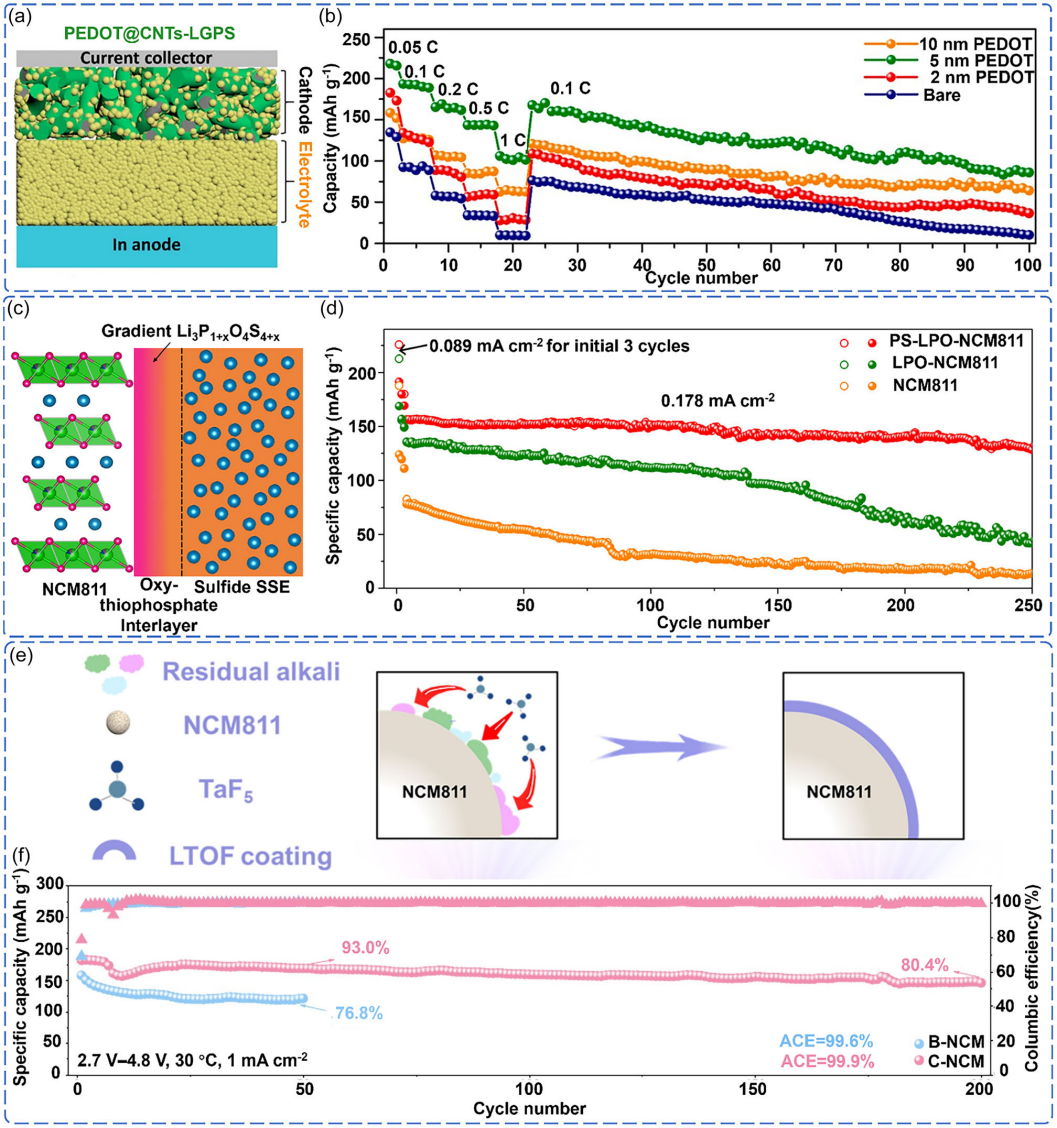
a) Schematic of the designed model cells. b) Rate capability and cycling stability of ASSLB employing NCM811 cathodes with different thicknesses of PEDOT coating. Reproduced with permission.^[^ ^94^ ^]^ Copyright 2020, American Chemical Society. c) Schematic of a gradient Li‐*P*‐O‐S coating at the NCM811/sulfide SSE interface. d) Cycling performance of ASSLB with three cathodes. Reproduced with permission.^[^ ^66^ ^]^ Copyright 2023, Springer Nature. e) Schematic of the in situ gas‐solid synthesis process for the Li‐Ta‐O‐F (LTOF) oxyhalide coating. f) Long‐term cycling performance of ASSLB employing bare NCM811 and LTOF‐coated NCM811 cathodes. Reproduced with permission.^[^ ^95^ ^]^ Copyright 2024, Wiley‐VCH.

Among the recent advances in coating materials and synthesis strategies, several promising approaches have emerged. To address the issue of carbon additives providing sufficient electron conduction pathways in the composite cathode, which may lead to severe side reactions and promote the decomposition of sulfide SSE, Deng et al. employed molecular layer deposition (MLD) technology to fabricate a PEDOT semiconductor film on the surface of NCM811 (**Figure** **6**a). This film acts as an interface protective layer, replacing the electrochemically reactive carbon additives on the NCM811 surface, thereby effectively suppressing the side reactions between the electrolyte and the cathode. The modified ASSLB achieved a reversible capacity of 102 mAh g^−1^ at a 1 C rate, significantly outperforming the untreated system (Figure 6b).^[^ ^94^ ^]^ This study demonstrates that semiconductor polymer coatings on the cathode surface can effectively enhance the interface stability of ASSLB.

Compared to traditional monolayer coatings, gradient structures, through continuous regulation of components and structures, can effectively address multiple degradation mechanisms. Liang et al. employed atomic layer deposition (ALD) technology to prepare Li_3_P_1+*x*_O_4_S_4*x*_ coatings with a sulfur content gradient, where the sulfur concentration gradually increases from the inner to outer layers. The outer sulfide layer's chemical properties closely resemble those of sulfide SSE, effectively hindering the structural degradation associated with the layered‐to‐spinel transformation in the grain boundaries and stabilizing the cathode/SSE interface during cycling (Figure 6c).^[^ ^66^ ^]^ The modified ASSLB maintained 80% capacity retention after 250 cycles at a cutoff voltage of 4.3 V and a current density of 0.178 mA cm^−2^ (Figure 6d). This successful gradient design represents a shift from “discrete modification” to “continuous interfacial regulation,” offering a new direction for improving interfacial compatibility.

Emerging synthesis strategies provide greater control over coating structures and performance. For example, Liu et al. utilized an in situ gas–solid reaction with TaF_5_ additives and the LiOH and Li_2_CO_3_ residues on the surface of NCM811 to form a uniform Li–Ta–O–F halogen oxide coating (Figure 6e).^[^ ^95^ ^]^ This coating achieved improved high‐voltage stability (maintaining 94% capacity after 500 cycles at 4.5 V, 1 mA cm^−2^) and highlighted the critical role of synthesis conditions in determining coating performance (Figure 6f). Similarly, Kim et al. developed a flash‐light sintering (FLS) to rapidly form a uniform and dense lithium lanthanum titanate (LLTO) protective layer on the NCM811 surface through millisecond‐pulse light irradiation.^[^ ^96^ ^]^ Compared to traditional high‐temperature sintering, FLS completes the sintering in a very short time, avoiding cation disordering and secondary phase formation, while achieving full coverage of the coating. This technique enabled the ASSLB to achieve a high initial capacity of 157.38 mAh g^−1^ at a 1 C rate, with 95% capacity retention after 50 cycles. This new process offers a promising new route for scalable production. Through precise control of reaction kinetics and thermodynamic processes, these innovative synthesis methods provide potential pathways toward achieving uniform and high‐quality protective layers.

Although significant progress has been made in the aforementioned studies, coating strategies that balance both performance and practicality still face many challenges. Current coating designs often require trade‐offs between competing performance metrics, such as mechanical flexibility and chemical stability, ionic conductivity and electronic insulation, process simplicity, and coating uniformity. Therefore, addressing these fundamental issues requires an in‐depth understanding of the atomic and molecular interactions between the coating and substrate, the interfacial ion migration mechanisms, and the long‐term electrochemical degradation paths.

Scalability and cost‐effectiveness remain key challenges for coating technologies in commercial applications. While laboratory‐scale results are promising, industrialization still faces challenges such as controlling coating thickness, ensuring large‐area uniformity, and achieving compatibility with existing manufacturing processes. For instance, the practical feasibility of ALD coatings requires careful evaluation. The slow deposition rate, high precursor and vacuum equipment costs, and challenges in scaling up powder ALD processes make it cost‐prohibitive for mass production, despite its precision and uniformity. Cost‐effective alternatives, such as scalable wet‐chemical methods or faster techniques like MLD, should be further explored for industrial applications. Additionally, ensuring process consistency and repeatability is crucial for meeting mass production requirements. Therefore, advancing the commercialization of coating technologies will require both comprehensive fundamental research and the collaborative development of application processes. While these advances represent important progress, significant challenges remain in translating laboratory successes to practical applications, particularly regarding long‐term stability and scalability.

### 3.2 Bulk Structure Engineering of NCM811

Although surface coating provides direct interfacial protection, bulk‐phase doping fundamentally enhances the interfacial stability between NCM811 and sulfide SSE through atomic‐scale structural modifications (Table S2, Supporting Information). This strategy relies on a multi‐scale synergistic effects: it stabilizes the crystal structure at the atomic level, regulates charge distribution at the electronic structure level, and improves chemical and electrochemical compatibility at the interface, thereby offering a systematic solution to address multiple failure mechanisms.

**Figure 7:**
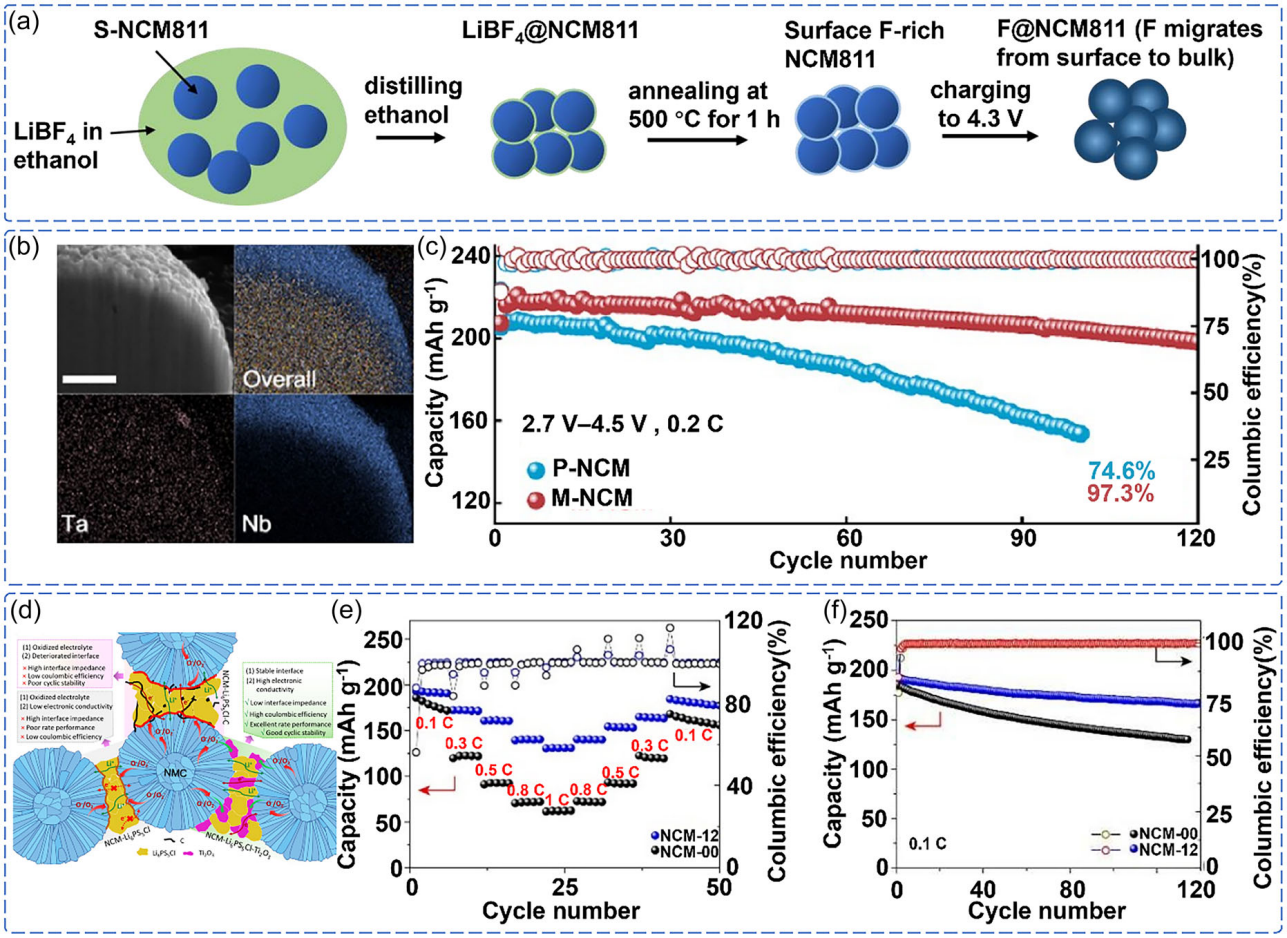
a) Schematic of the formation of F‐doped NCM811. Reproduced with permission.^[^ ^97^ ^]^ Copyright 2023, Springer Nature. b) Cross‐sectional SEM and elemental mapping images of Ta/Nb‐doped NCM811. c) Cycling performance of mold ASSLB with pristine NCM811 and Ta/Nb‐doped NCM811. Reproduced with permission.^[^ ^98^ ^]^ Copyright 2024, American Chemical Society. d) Schematic illustration of interfacial structures in NCM‐00, NCM‐C, and NCM‐12 composite cathodes. e) Rate capability of ASSLB at 35 °C. f) Long‐term cycling performance of ASSLB at 0.1 C. Reproduced with permission.^[^ ^99^ ^]^ Copyright 2023, Elsevier.

Recent advancements in halogen element doping have demonstrated significant interfacial stability effects. Wan et al. revealed a new dynamic stabilization mechanism through F doping, where F^−^ undergoes electrochemical migration during charging to 4.3 V, diffusing from the NCM811 surface into the bulk phase to form a uniform F^−^‐doped bulk structure (**Figure** **7**a).^[^ ^97^ ^]^ This migration behavior effectively suppresses crack formation during cycling by enhancing the integrity of the cathode structure. This study indicates that dopants with migration capability hold great promise for constructing adaptive interface protective layers, offering new avenues for dynamic interface regulation.

Multielement doping strategies offer potential advantages in regulating complex interfacial properties. Dai et al. developed a Ta‐ and Nb‐based competitive diffusion system that achieved a dual stability mechanism: Ta‐O bonds help to inhibit excessive oxidation of lattice oxygen, while the LiNbO_3_ interfacial layer induced by Nb optimizes the Li^+^ transport path through a polarized electric field (Figure 7b).^[^ ^98^ ^]^ The designed NCM811‐sulfide SSE ASSLB exhibited improved cyclability under 4.5 V (97.3% after 120 cycles) (Figure 7c). This result shows that precise control of multielement diffusion dynamics can simultaneously address multiple challenges such as bulk phase structure degradation and interfacial mismatch, providing a novel strategy for high‐voltage ASSLB design. In a deep exploration of doping e‐, Fang et al. investigated the doping mechanism of Ti_2_O_3_‐based dopant materials with high electronic conductivity (10–10^2^ S cm^−1^) that can replace carbon additives.^[^ ^99^ ^]^ They discovered that Ti^3+^ serves a dual function: not only capturing lattice oxygen to suppress structural instability but also enhancing electronic conductivity. This system achieved 86.5% capacity retention (140 cycles) at a 4.3 V cutoff voltage, highlighting the application potential of multifunctional dopants (Figure 7d–f). However, the observed performance degradation under higher voltages indicates that further exploration of the stability boundaries and mechanisms of dopants under extreme conditions is still needed.

The strategy of using bulk‐phase doping to regulate interfacial stability is evolving from traditional single‐element doping toward more complex approaches involving multielement synergy, integration of functional materials, and dynamic migration processes. However, these doping strategies face substantial challenges in practical applications that limit their effectiveness. The primary issue is ensuring dopant stability under harsh operating conditions, such as high voltage and high current density, as evidenced by the performance degradation of Ti_2_O_3_ systems under extreme conditions. Additionally, precisely controlling the complex interfacial reaction dynamics, particularly the interactions between dopants, cathode, and sulfide SSE, presents significant technical challenges. Furthermore, the coupling behavior between bulk‐phase structural evolution and interfacial stability remains poorly understood and is crucial for achieving long‐term durability. These fundamental limitations suggest that while bulk doping can provide improvements, it cannot entirely address the complex degradation mechanisms identified in Section 2, highlighting the need for continued research into the fundamental interactions between dopant distribution, bulk structural integrity, and interfacial activity.

### 3.3 Synergistic Modification Strategies of NCM811

Although individual surface coating or bulk‐phase doping strategies have improved interfacial stability to some extent, recently emerging synergistic modification strategies systematically address multiple interfacial failure mechanisms, providing effective approaches to further enhance interfacial stability.^[^ ^100^ ^]^ This strategy, which combines highly ion‐conductive surface coatings with bulk‐phase doping for crystal structure stabilization, exhibits excellent interfacial regulation capabilities in constructing high‐performance NCM811‐sulfide SSE‐based ASSLB.^[^ ^101^ ^]^

**Figure 8:**
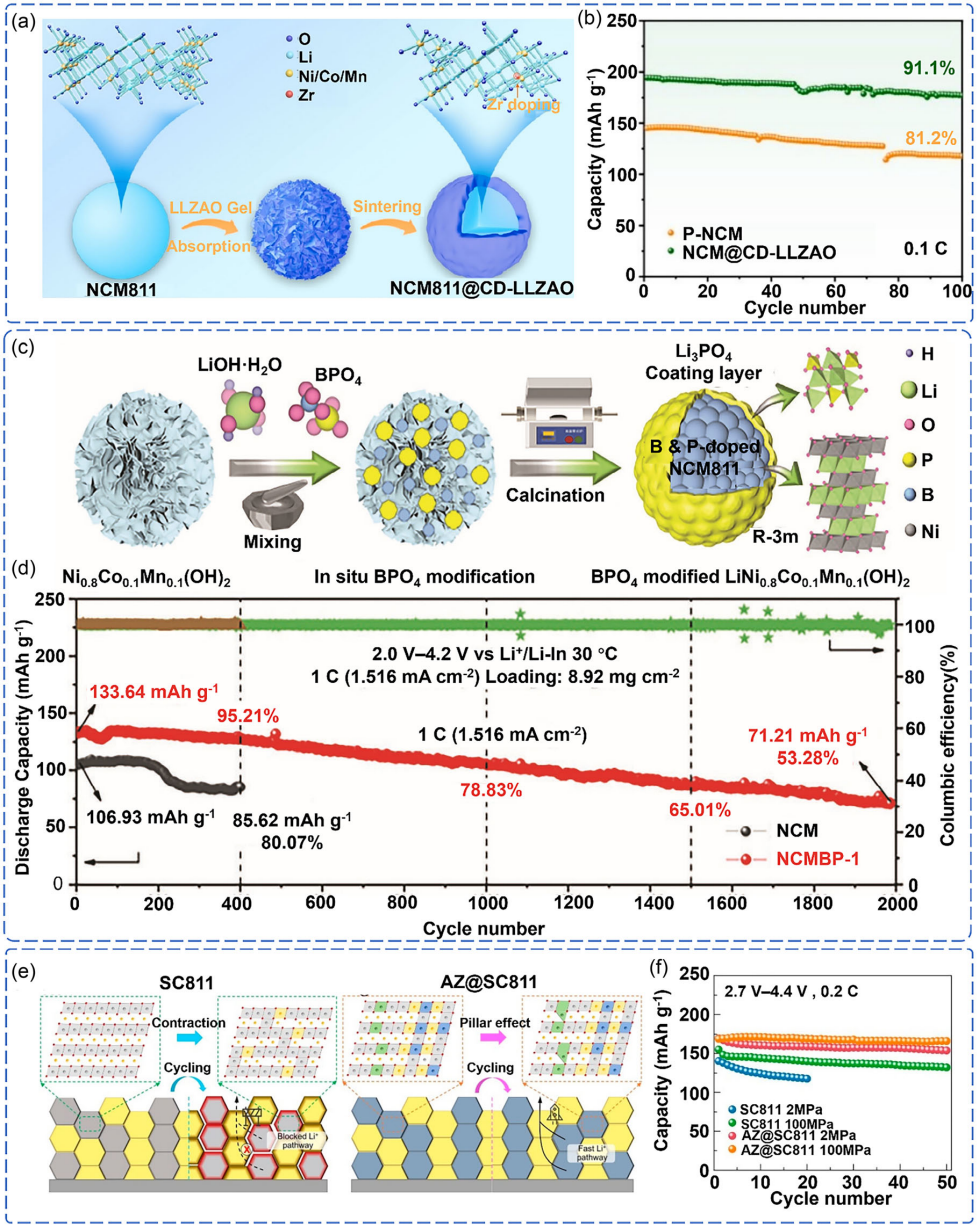
a) Schematic of the synthesis process of dual‐functional modified NCM811. b) Cycling performance of the pristine NCM811 and NCM811@CD‐LLZAO. Reproduced with permission.^[^ ^102^ ^]^ Copyright 2024, Elsevier. c) Cross‐sectional SEM and elemental mapping images of pristine NCM811. d) Cycling performance of ASSLB with pristine NCM811 and modified NCM811. Reproduced with permission.^[^ ^103^ ^]^ Copyright 2023, Wiley‐VCH. e) Structural evolution of SC811 and AZ@SC811 composite cathodes during cycling. f) Cycling performance of SC811 and AZ@SC811 under different stacking pressures. Reproduced with permission.^[^ ^105^ ^]^ Copyright 2023, American Chemical Society.

Li et al. demonstrated a synergistic modification strategy involving Zr‐doped bulk phase and Li_6.25_La_3_Zr_2_Al_0.25_O_12_ (LLZAO) coating (NCM811@CD‐LLZAO).^[^ ^102^ ^]^ The LLZAO coating provides high ionic conductivity and electronic insulation at the surface, while the Zr–O bonding stabilizes the bulk lattice structure (**Figure** **8**a). This synergistic design achieves excellent performance, maintaining 91.1% of the capacity after 100 cycles at 0.1 C (Figure 8b). This surface‐bulk phase synergistic modification strategy paves a new way for the practical application of high‐energy‐density ASSLB.

In addition to electrochemical stability, synergistic modification of dopants and coatings has also significantly improved high‐voltage operation interface stability. Shi et al. significantly improved interfacial stability under high‐voltage operation using a Li_3_PO_4_ coating and B/P codoping.^[^ ^103^ ^]^ The Li_3_PO_4_ coating suppresses anion exchange behavior at the interface, effectively inhibiting side reactions and improving interface stability, while B^3+^/P^5+^ codoping enhances lattice oxygen stability in NCM811 and the integrity of its layered structure through strong B‐O and P‐O bonds (Figure 8c). ASSLB employing this strategy delivered excellent cycling stability at high voltages (≥4.6 V vs Li^+^/Li), with a capacity fade rate of only 0.01% per cycle after 2300 cycles at 1 C, significantly lower than that of unmodified electrodes (Figure 8d). To demonstrate the broader applicability of this approach, the team later developed a modification scheme based on Li_7_TaO_6_ coating and Ta^5+^ bulk‐phase doping.^[^ ^104^ ^]^

Furthermore, synergistic modification also plays a crucial role in ensuring the mechanical stability required for practical applications. Zhao et al. developed a strategy using a surface Li_2_ZrO_3_ coating and bulk‐phase Al doping (AZ@SC811) to address the mechanical reliability challenges of ASSLB under high stacking pressures.^[^ ^105^ ^]^ This strategy regulates stress distribution across the entire cathode/SSE interface, effectively alleviating mechanical damage during cycling (Figure 8e). At just 2 MPa of stacking pressure, this battery system maintained 96.27% capacity retention after 1000 cycles at 2 C (Figure 8f). This result demonstrates the practical application potential of surface‐bulk phase synergistic modification at the engineering level.

Recent advances introduce functional ionic conductors as interfacial bridges to reconstruct the interfacial microenvironment. Ye et al. introduced the halide Li_3_InCl_6_ (LIC) into the NCM811 composite cathode via scalable slurry‐coating, where LIC acts as a dual‐functional mediator.^[^ ^106^ ^]^ Unlike traditional conductive additives, LIC functions as a dual‐functional mediator through physical barrier effects and electrochemical stabilization. It prevents direct NCM811‐Li_5.5_PS_4.5_Cl_1.5_ contact to suppress parasitic reactions while utilizing its wide electrochemical window to inhibit SCL formation and by‐product generation. Although LIC exhibits lower ionic conductivity than conventional sulfide electrolytes, its high chemical stability and wide electrochemical window effectively suppress interfacial side reactions and the formation of SCL, thereby significantly enhancing interfacial compatibility, as demonstrated by a capacity retention of 77.4% after 100 cycles. This study highlights a design strategy that prioritizes interfacial stability over bulk ionic conductivity, offering new insights into addressing interfacial challenges associated with high‐voltage cathodes.

A summary of typical surface‐bulk phase synergistic modification methods for NCM811 is shown in Table S3, Supporting Information. The surface‐bulk phase synergistic modification strategy not only effectively addresses interfacial electrochemical stability, mechanical integrity, and structural retention but also provides the theoretical foundation and practical pathway for the structural‐function integrated design of ASSLB interface regulation methods. The success of various coating‐doping combinations demonstrates that well‐orchestrated synergistic modifications can effectively mitigate multiple degradation mechanisms simultaneously. However, these synergistic approaches still face significant challenges in large‐scale practical applications, including controlling doping concentration, regulating coating thickness, and ensuring thermodynamic compatibility at interfaces. Moreover, future development of synergistic strategies should focus on rational design principles that maximize synergistic effects while maintaining practical feasibility for large‐scale applications.

### 3.4 Sulfide SSE Optimization

To address interfacial instability in NCM811‐sulfide SSE‐based ASSLB, systematic research has been conducted along two avenues: NCM811 cathode modification and sulfide SSE optimization (Table S4, Supporting Information). In addition to traditional surface coating and bulk‐phase doping strategies for NCM811, recent efforts have increasingly focused on stabilizing the interface by modulating the intrinsic properties of sulfide SSE, which has become an important research direction. This approach encompasses electrolyte compositional engineering, crystal structure regulation, and the exploration of interface self‐healing mechanisms, and represents significant progress toward the practical application of high‐voltage ASSLB.

**Figure 9:**
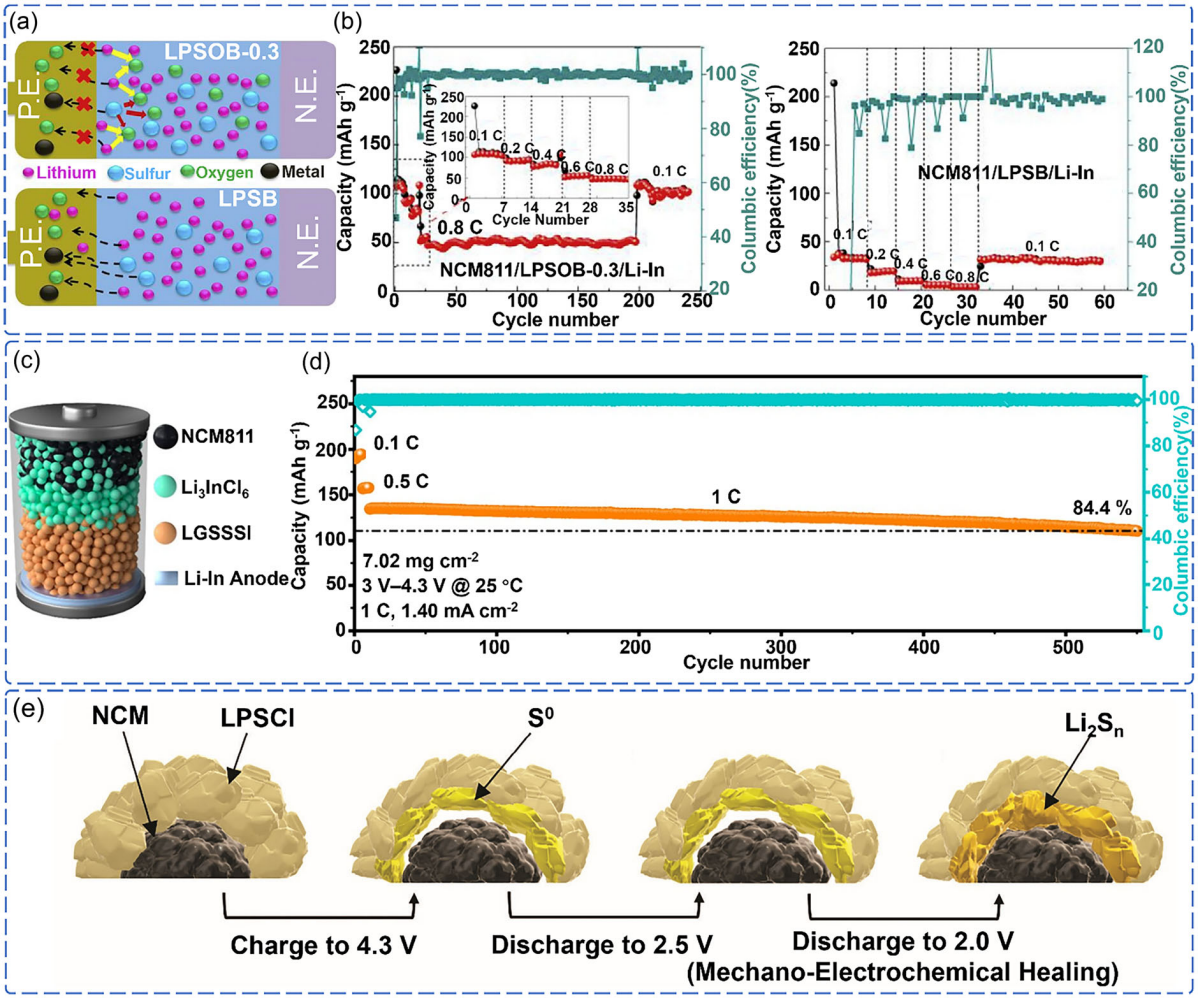
a) Schematic illustration of the advantage of oxygen doping in sulfide SSE. b) Cycling and rate performance of Li_6_PS_4.7_O_0.3_Br and Li_6_PS_5_Br. Reproduced with permission.^[^ ^77^ ^]^ Copyright 2019, Elsevier. c) Schematic of ASSLB with a gradient sulfide SSE structure. d) Long‐term cyclability of ASSLB employing the modified gradient sulfide SSE. Reproduced with permission.^[^ ^107^ ^]^ Copyright 2025, American Chemical Society. e) Schematic of the coupled mechano‐electrochemical self‐healing process at NCM811/Li_6_PS_5_Cl interface during cycling between 2.0–4.3 V (vs Li^+^/Li). Reproduced with permission.^[^ ^84^ ^]^ Copyright 2025, Wiley‐VCH.

In terms of optimizing the chemical composition of sulfide SSE, researchers have developed multiscale interface optimization strategies. Zhang et al. employed an oxygen‐doping strategy by partially replacing sulfur sites in Li_6_PS_5_Br with oxygen, successfully preparing Li_6_PS_4.7_O_0.3_Br electrolyte (**Figure** **9**a).^[^ ^77^ ^]^ This modification effectively inhibited interfacial cation migration and elemental interdiffusion and significantly suppressed SCL formation, thereby enhancing overall interface compatibility and the electrolyte's oxidation stability. The modified ASSLB exhibited a cycle life exceeding 92 cycles under identical conditions, significantly outperforming the unmodified system, which failed during the second charging cycle. Multi‐component synergistic doping also presents a promising approach for optimizing electrolyte lattice dynamics (Figure 9b). Ma et al. introduced codoping of Ge, Si, and Sb into Li_6_PS_5_I to replace P sites, enhancing the disorder of the S^2−^/I^−^ sites, thereby increasing the configurational entropy and reducing the activation energy for Li^+^ migration.^[^ ^107^ ^]^ More importantly, a gradient electrolyte structure was constructed by introducing a halide Li_3_InCl_6_ intermediate layer on the cathode side, which effectively suppressed direct contact and side reactions between NCM811 and sulfide SSE through both physical isolation and chemical passivation by the layer (Figure 9c). This design enabled ASSLB to achieve excellent cycling stability over a wide temperature range (–20 to 60 °C) and at high cathode loadings (100 mg cm^−2^), with a capacity retention of 84.4% after 550 cycles at a 1 C rate (Figure 9d).

Notably, sulfide electrolytes exhibit unique characteristics such as interfacial contact self‐recovery and stress buffering. Lee et al. found that in the NCM811+Li_6_PS_5_Cl+C/Li_6_PS_5_Cl/Li ASSLB system, within the 2.0–4.3 V (vs Li^+^/Li), the elemental sulfur (S^0^) generated upon oxidation of Li_6_PS_5_Cl enables dynamic reconstruction of the interfacial contact through moderate volume expansion of the lithiation products formed during discharge (Figure 9e).^[^ ^84^ ^]^ This coupled mechano‐electrochemical self‐healing mechanism provides a new approach to enhance the cycling reliability of ASSLB.

The above research demonstrates that directional regulation of the intrinsic properties of sulfide SSE can effectively improve its interface compatibility with NCM811 high‐voltage cathodes. Future research should further explore the composition‐structure‐performance relationships, while simultaneously optimizing both the electrolyte's intrinsic properties and interface stability, providing crucial theoretical insights and breakthroughs for the practical and commercial deployment of NCM811‐sulfide SSE‐based ASSLB.

## 4 Conclusion and Outlook

**Figure 10:**
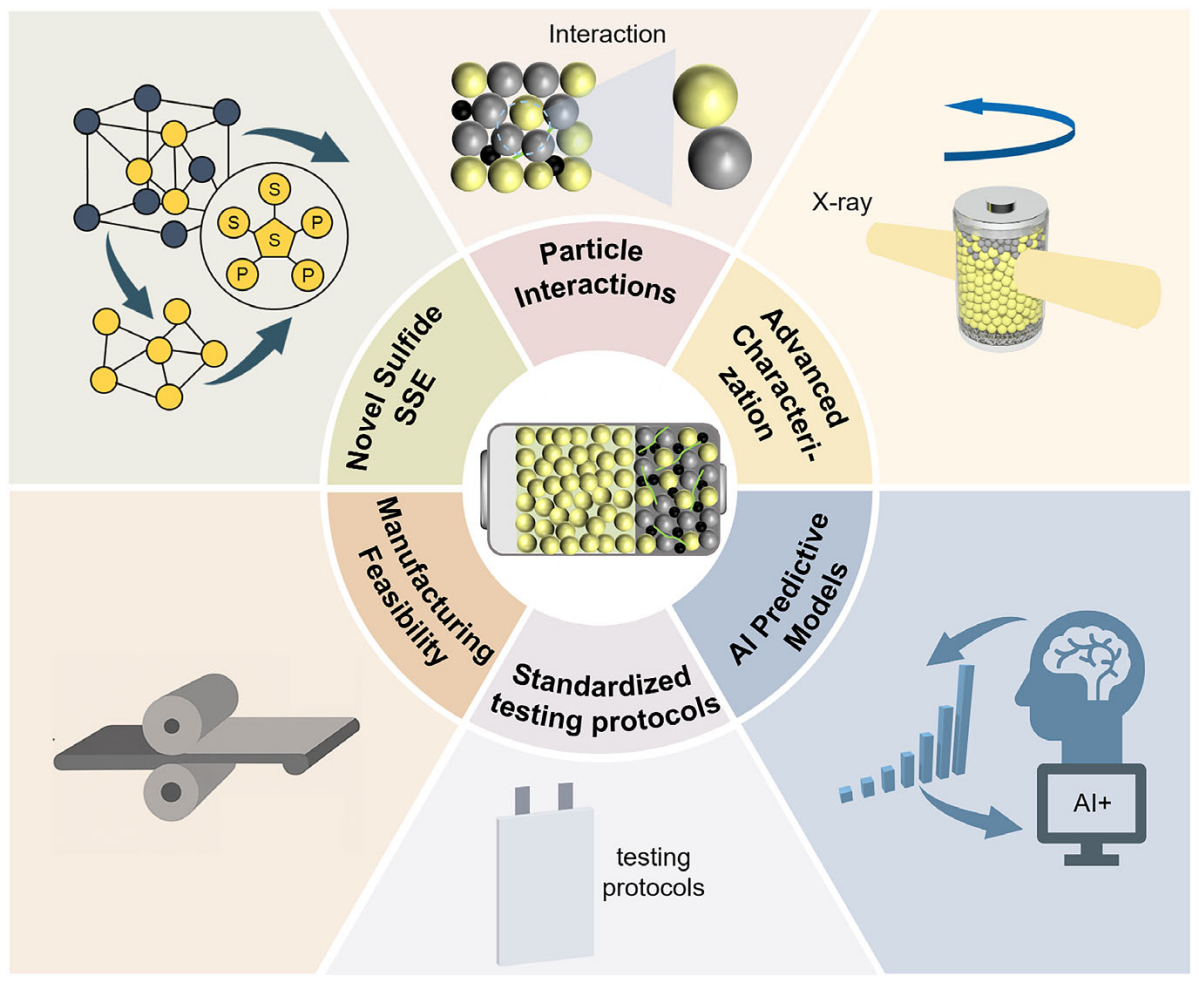
Key research directions for NCM811–sulfide SSE‐based ASSLB.

In summary, NCM811‐sulfide SSE‐based ASSLB, offering high energy density and excellent intrinsic safety, represents a significant direction for next‐generation energy storage. However, their practical deployment is hindered by coupled multimechanism deterioration at the cathode/SSE interface. This degradation manifests through three interconnected pathways. Mechanical contact degradation occurs when anisotropic particle volume changes lead to microcrack initiation, resulting in ion channel blockage and consequently local current concentration. Chemical interface degradation follows as local current concentration generates a high electrochemical potential gradient, which lowers the reaction energy barrier and accelerates side reactions and SCL formation. Electrochemical performance degradation emerges when side reaction products and SCL increase interface impedance, leading to intensified polarization and capacity decay, while high‐voltage operation further exacerbates mechanical stress concentration. These three mechanisms form a self‐reinforcing degradation cycle where mechanical damage exposes fresh interfaces, chemical reactions accelerate at these interfaces, electrochemical performance deteriorates, and increased mechanical stress perpetuates the cycle. While existing research on NCM811 surface engineering, bulk‐phase doping, surface‐bulk synergistic modification, and interface‐oriented modification of sulfide SSE has reduced interface impedance and delayed capacity fade, it remains insufficient to eliminate the dynamic degradation of interfacial chemical reactions, increasing impedance, and contact deterioration during cycling. The root cause is the inherent thermodynamic incompatibility between NCM811 and sulfide SSE, which continuously drives decomposition reactions and mechanical mismatch, thereby perpetuating dynamic degradation. Although this theoretical framework helps identify the coupling relationships between different mechanisms, the dynamic coupling across different time scales and their quantitative interactions are not yet fully resolved. Furthermore, to achieve the commercialization goals of high energy density and long cycle life in ASSLB, future research should prioritize breakthroughs in the following six critical areas (**Figure** **10**): 1) Design of intrinsically stable sulfide SSE: Current sulfide SSE exhibit narrow electrochemical stability windows, undergoing oxidative decomposition at high voltages that create insulating phases and compromise battery performance. This fundamental limitation stems from the inherent chemical nature of sulfide frameworks and requires addressing stability at multiple levels. Among potential approaches, molecular structure engineering offers the most promising pathway by modifying bonding environments and electronic structures to enhance oxidation resistance, while crystal structure optimization can create protective coordination environments and alter decomposition pathways. Additionally, interface engineering and compositional gradients provide complementary strategies, though fundamental stability improvements must ultimately originate from intrinsic structural modifications that preserve ionic conductivity while expanding electrochemical windows.^[^ ^108^ ^]^ 2) Optimizing particle interactions: Contemporary ASSLB commercialization efforts focus primarily on achieving compatibility with high‐voltage cathodes such as NCM811, yet the particle interfacial challenges become significantly amplified under these demanding conditions. The higher operating voltages and increased reactivity of high‐nickel cathodes exacerbate inter‐particle mechanical coupling issues, intensify cathode particle–electrolyte particle reactions that generate lattice oxygen and trigger sulfide decomposition cycles, and accelerate interfacial degradation processes. Future research should prioritize developing robust particle interfacial engineering strategies specifically designed for high‐voltage cathode compatibility, with emphasis on creating chemically inert yet highly conductive interparticle layers that can withstand the harsh electrochemical environment while maintaining efficient ion and electron transport pathways. 3) Advanced characterization techniques: Traditional destructive testing methods are limited in capturing the dynamic evolution of the buried cathode/SSE interface, highlighting the urgent need for advanced in situ characterization.^[^ ^109^, ^110^ ^]^ While individual operando techniques such as transmission X‐ray microscopy, X‐ray absorption spectroscopy (XAS), and coherent diffraction imaging have been demonstrated, the critical challenge lies in establishing systematic correlations between chemical reactions, mechanical stress evolution, and morphological changes under realistic electrochemical conditions. Future efforts should focus on developing integrated multimodal characterization platforms that simultaneously monitor these three aspects during battery operation. This systematic chemical–mechanical–morphological mapping would provide mechanistic insights into how current optimization strategies—surface coatings reducing initial impedance, doping modifying bulk properties, and interface engineering improving contact quality—address different aspects of the degradation cascade, thereby guiding the development of more targeted solutions that simultaneously address multiple degradation mechanisms.^[^ ^111^, ^112^, ^113^ ^]^ 4) Developing AI‐enhanced predictive degradation models: Current ASSLB research lacks predictive capability to forecast long‐term performance under real‐world conditions. Integrating machine learning algorithms with physics‐based degradation models that capture chemical kinetics, mechanical deformation, and electrochemical transport enables quantitative prediction of capacity fade, impedance growth, and failure modes across different operating conditions. AI‐driven models can incorporate stochastic elements to account for manufacturing variability, learn from experimental datasets to refine predictive accuracy, and provide probabilistic lifetime predictions essential for commercial deployment. This AI‐enhanced predictive framework guides accelerated testing protocols and enables rational design optimization. 5) Developing standardized testing protocols: Performance evaluation of ASSLB currently faces significant challenges. Substantial variations in testing environments, stacking pressure control, and electrode parameters between laboratories severely limit data comparability and reproducibility. To enable reliable cross‐study comparisons and accelerate industrialization, a unified standardized testing protocol ensuring data consistency is imperative. Recent studies propose a preliminary framework covering key aspects, including pressure control; initial open‐circuit voltage screening criteria; standardized current densities and electrode loadings; material pretreatment procedures; and requirements for conducting at least three replicate experiments with reporting of cell failure rates to quantify batch consistency.^[^ ^54^, ^114^, ^115^ ^]^ Future efforts should establish an authoritative, widely adopted standardized test system. 6) Enhancing manufacturing feasibility: Sulfide SSE faces two critical commercialization barriers. First, extreme moisture sensitivity necessitates costly inert atmosphere processing and stringent environmental controls. Second, material costs exceed liquid electrolytes by over 100‐fold, creating prohibitive economic barriers. Additionally, limited shelf life complicates supply chain management and increases waste. Breakthrough solutions must develop moisture‐stable sulfide compositions enabling ambient processing, establish cost‐effective synthesis routes using abundant precursors rather than expensive lithium sulfide, and design manufacturing processes that extend material stability while reducing environmental control requirements.

Despite the significant fundamental and technical challenges facing current NCM811–sulfide SSE‐based ASSLB, an in‐depth understanding of solid‐state electrochemistry and solid‐solid electrode/electrolyte interface mechanisms, coupled with future key technological breakthroughs, is expected to pave the way for their gradual large‐scale commercial deployment.

## Conflict of Interest

The authors declare no conflict of interest.

## Supporting information

Supplementary Material
