# Supplementary material for: NCM811–Sulfide Electrolyte Interfacial Degradation Mechanisms and Regulation Strategies in All‐Solid‐State Lithium Battery
Source: ChemSusChem. 2025 Oct 13;18(23):e202501033. doi: 10.1002/cssc.202501033 (PMC12665888; doi:10.1002/cssc.202501033)
Supplement: Supplementary file 1 — Supplementary Material [file CSSC-18-e202501033-s001.pdf]

NCM811-Sulfide Electrolyte Interfacial Degradation Mechanisms and Regulation Strategies in All-Solid-State Lithium Battery

Haoyu Feng,[a] Guanghan Zhu,*[b] Ziming Wan,[a] Feng Ryan Wang,[b] Zhangxiang Hao,*[a] and Junrun Feng*[a]

[a] H. Feng, Z. Wan, Z. Hao, J. Feng
School of Science, School of Chip Industry
Hubei University of Technology
Wuhan 430068, China
E-mail: haozx@hbut.edu.cn
fengjunrun@hbut.edu.cn

[b] G. Zhu, F. R. Wang
Materials and Catalysis Laboratory, Department of Chemical Engineering
University College London
London WC1E 7JE, United Kingdom
E-mail: guanghan.zhu@ucl.ac.uk

Table S1. Performance summary of NCM811–sulfide SSE-based ASSLB modified by NCM811 surface coating.

Cathode/electrolyte/anode	Coating	Voltage range (V vs Li^+/Li)	Tempe-rature	Capacity (mAh g^{-1})	Cycle Performance	Ref.
$LiNbO_3$@NCM811+$Li_{10}GeP_2S_{12}$ (73:27)/$Li_{10}GeP_2S_{12}$/Li-In	$LiNbO_3$	2.7–4.38 V	35 °C	162 (20 mA g^{-1})	77.9% (100 mA g^{-1}, 50 cycles)	[116]
		2.7–4.38 V	60 °C	203 (20 mA g^{-1})	55.2% (100 mA g^{-1}, 50 cycles)	
$LiCoO_2$@NCM811+$Li_{10}GeP_2S_{12}$ (73:27)/$Li_{10}GeP_2S_{12}$/Li-In	$LiCoO_2$	2.7–4.38 V	35 °C	182.4 (20 mA g^{-1})	80% (60 mA g^{-1}, 585 cycles)	[82]
$LiNbO_3$@NCM811+$Li_{10}GeP_2S_{12}$ (73:27)/$Li_{10}GeP_2S_{12}$/Li-In	$LiNbO_3$	2.7–4.38 V	35 °C	134.6 (60 mA g^{-1})	68.8% (60 mA g^{-1}, 100 cycles)	
$LiCoO_2$-$LiNbO_3$@NCM811+$Li_{10}GeP_2S_{12}$ (73:27)/$Li_{10}GeP_2S_{12}$/Li-In	$LiCoO_2$-$LiNbO_3$	2.7–4.38	35 °C	182.4 (20 mA g^{-1})	80% (60 mA g^{-1}, 585 cycles)	
Li_3PO_4@NCM811+$Li_{10}GeP_2S_{12}$ (70:30)/$Li_{10}GeP_2S_{12}$/Li-In	Li_3PO_4	2.7–4.5 V	RT	170.6 (0.1 C)	58.9% (0.2 C, 300 cycles)	[98]
Li_2O@NCM811+Li_6PS_5Cl (70:30)/Li_6PS_5Cl/Li-In	Li_2O	2.6–4.2V	30 °C	170.81 (17 mA g^{-1})	94.6% (170 mA g^{-1}, 1290 cycles)	[96]
	Li_2O	2.6–4.2 V	30 °C	189.66 (17 mA g^{-1})	81.5% (170 mA g^{-1}, 2581 cycles)	
Li_2O@NCM811+$Li_{9.54}Si_{1.74}P_{1.44}S_{11.7}Cl_{0.3}$ (70:30)/$Li_{9.54}Si_{1.74}P_{1.44}S_{11.7}Cl_{0.3}$/Li-In	Li_2O	2.72–4.4 V	25 °C	137 (20 mA g^{-1})	100% (200 mA g^{-1}, 500 cycles)	[117]
	Li_2O	2.72–4.5 V	25 °C	143 (20 mA g^{-1})	100.10% (200 mA g^{-1}, 200 cycles)	
$Li_{10}SnP_2S_{12}$-Li_2ZrO_3@NCM811+Li_6PS_5Cl (70:29)/Li_6PS_5Cl/Li-In	$Li_{10}SnP_2S_{12}$-Li_2ZrO_3	2.5–4.25 V	30 °C	175 (90 mA g^{-1})	71.4% (90 mA g^{-1}, 500 cycles)	[118]
LiOH@NCM811+Li_6PS_5Cl (70:30)/Li_6PS_5Cl/Li-In	LiOH	2.5–4.2 V	RT	158.87 (20 mA g^{-1})	90% (20 mA g^{-1}, 600 cycles)	[97]
$LiNbO_x$@NCM811+$Li_{10}GeP_2S_{12}$ (65:30)/$Li_{10}GeP_2S_{12}$/Li-In	$LiNbO_x$	2.6–4.4 V	RT	160 (0.1 C)	61.9% (0.1 C,50 cycles)	[119]
Li-Ta-O-F@NCM 811+Li_6PS_5Cl (70:28)/Li_6PS_5Cl/Li-In	Li-Ta-O-F	2.7–4.5 V	RT	151.5 (22 mA g^{-1})	94% (22 mA g^{-1}, 500 cycles)	[88]

	Li-Ta-O-F	2.7–4.8 V	RT	181.8 (22 mA g^{-1})	80.4% (22 mA g^{-1}, 200 cycles)	
$Li_3P_{1+x}O_4S_{4x}$@NCM811+$Li_{10}GeP_2S_{12}$ (70:30)/$Li_{10}GeP_2S_{12}$/Li-In	$Li_3P_{1+x}O_4S_{4x}$	2.7–4.3 V	25 °C	161 (0.178 mA cm^{-2})	79.5% (0.178 mA cm^{-2},250 cycles)	[66]
LLTO@NCM 811+Li_6PS_5Cl (60:35)/Li_6PS_5Cl/Li-In	LLTO	2.5–4.25 V	45 °C	164 (1 C)	95% (1 C,50 cycles)	[103]
B_2O_3/$LiBO_2$@NCM811+Li_6PS_5Cl (70:27)/Li_6PS_5Cl/Gr+Li_6PS_5Cl (60:40)	B_2O_3/ $LiBO_2$	2.8–4.3 V	25 °C	120.9 (36 mA g^{-1})	84.19% (36 mA g^{-1}, 500 cycles)	[119]
LNO@NCM811+$Li_{10}SnP_2S_{12}$ (50:46)/$Li_{10}SnP_2S_{12}$/$Li_4Ti_5O_{12}$ + $Li_{10}SnP_2S_{12}$ (50:46)	LNO	2.85–4.35 V	25 °C	205.4 (18 mA g^{-1})	90.3% (54 mA g^{-1}, 100 cycles)	[120]
Li_2SiO_x@NCM811+Li_6PS_5Cl (77:19)/Li_6PS_5Cl/Si+Li_6PS_5Cl (60:30)	Li_2SiO_x	2.4–4.2 V	RT	145 (67 mA g^{-1})	62.9% (67 mA g^{-1},1000 cycles)	[99]
Al-GL@NCM811+$Li_{9.54}Si_{1.74}P_{1.44}S_{11.7}Cl_{0.3}$ (50:48)/$Li_{9.54}Si_{1.74}P_{1.44}S_{11.7}Cl_{0.3}$/ $Li_4Ti_5O_{12}$	Al-GL	2.8–4.3 V	30 °C	190.0 (18 mA g^{-1})	88.0% (36 mA g^{-1}, 100 cycles)	[122]
Al-GL@NCM811+$Li_{9.54}Si_{1.74}P_{1.44}S_{11.7}Cl_{0.3}$ (50:48)/$Li_{9.54}Si_{1.74}P_{1.44}S_{11.7}Cl_{0.3}$/Li-In	Al-GL	2.8–4.3 V	60 °C	155.3 (180 mA g^{-1})	80.0% (180 mA g^{-1}, 1000 cycles)	
FeO·Fe_2O_3@NCM811+Li_6PS_5Cl/Li_6PS_5Cl/$Li_4Ti_5O_{12}$+Li_6PS_5Cl	FeO·Fe_2O_3	2.65–4.35 V	RT	126 (40 mA g^{-1})	111.1% (40 mA g^{-1}, 100 cycles)	[123]
PEDOT@NCM811+$Li_{10}GeP_2S_{12}$ (65:30)/$Li_{10}GeP_2S_{12}$/$Li_4Ti_5O_{12}$+Li_6PS_5Cl	PEDOT	2.7–4.4 V	RT	100 (1 C)	51.1% (1 C, 100 cycles)	[87]
$LiAlO_2$@NCM811/$Li_{5.5}PS_{4.5}Cl_{1.5}$/Li	$LiAlO_2$	2.7–4.2 V	25 °C	139.46 (8.5 mA g^{-1})	82.4% (8.5 mA g^{-1}, 60 cycles)	[49]
Li_5FeO_4@NCM811+Li_6PS_5Cl (70:25)/Li_6PS_5Cl/In	Li_5FeO_4	2.5–4.3 V	RT	199.7 (17 mA g^{-1})	84.8% (34 mA g^{-1}, 100 cycles)	[102]
Li_3VO_4@NCM811+Li_6PS_5Cl (70:30)/Li_6PS_5Cl/Li-In	Li_3VO_4	2.8–4.5 V	RT	142.4 (200 mA g^{-1})	80.0% (200 mA g^{-1}, 2000 cycles)	[101]
Li_2ZrO_3@NCM811+$Li_{6.7}P_{0.3}Ge_{0.7}S_5I$	Li_2ZrO_3	3.0–4.2 V	25 °C	155.2 (0.2 C)	84% (0.2 C,	[100]

(70:30)/$Li_{6.7}P_{0.3}Ge_{0.7}S_5I$/Li-In					300 cycles)	
$Li_{1.3}Al_{0.3}Ti_{1.7}(PO_4)_3$@NCM811 +$Li_{5.5}PS_{4.5}Cl_{1.5}$ (70:30)/$Li_{5.5}PS_{4.5}Cl_{1.5}$/Li-In	$Li_{1.3}Al_{0.3}Ti_{1.7}(PO_4)_3$	2.7–4.2 V	25 °C	151.5 (20 mA g^{-1})	81.6% (20 mA g^{-1}, 300 cycles)	[124]

Table S2. Performance summary of NCM811–sulfide SSE-based ASSLB modified by NCM811 bulk doping.

Cathode/electrolyte/anode	Dopant	Voltage range （V vs Li^+/Li）	Tempe -rature	Capacity (mAh g^{-1})	Cycle Performance	Ref.
F@NCM811+Li_6PS_5Cl (50:46)/Li_6PS_5Cl-$Mg_{16}Bi_{84}$/Li	F	2.7–4.3 V	80 °C	185 (200/3 mA g^{-1})	80% (200/3 mA g^{-1}, 40 cycles)	[89]
Cl@NCM811+Li_6PS_5Cl (50:46)/Li_6PS_5Cl-$Mg_{16}Bi_{84}$/Li	Cl	2.7–4.1 V	80 °C	167 (600 mA g^{-1})	65% (600 mA g^{-1}, 300 cycles)	[89]
Ti_2O_3@NCM811+Li_6PS_5Cl (70:25)/Li_6PS_5Cl/Li	Ti_2O_3	2.7–4.3 V	35 °C	192 (20 mA g^{-1})	86.5% (20 mA g^{-1}, 140 cycles)	[91]
	Ti_2O_3	2.7–4.3 V	35 °C	153 (100 mA g^{-1})	75.2% (100 mA g^{-1}, 300 cycles)	
Ta, Nb@NCM811+$Li_{10}SnP_2S_{12}$ (70:30)/Li_6PS_5Cl/Li-In	Ta, Nb	2.7–4.5 V	25 °C	210.1 (44 mA g^{-1})	97.3% (44 mA g^{-1}, 120 cycles)	[90]

Table S3. Performance summary of NCM811–sulfide SSE-based ASSLB modified by combined NCM811 surface coating and bulk doping.

Cathode/electrolyte /anode	Coating	Dopant	Voltage range（V vs Li^+/Li）	Tempe -rature	Capacity (mAh g^{-1})	Cycle Performance	Ref.
NCM811+Li_6PS_5Cl (50:50)/Li_6PS_5Cl/Li-In	$Li_{6.25}La_3Zr_2Al_{0.25}O_{12}$	Zr^{4+}	2.8–4.3 V	RT	125 (180 mA g^{-1})	70.1% (180 mA g^{-1}, 1000 cycles)	[92]
	$Li_{6.25}La_3Zr_2Al_{0.25}O_{12}$	Zr^{4+}	2.8–4.3 V	RT	187.7 (18 mA g^{-1})	91.1% (18 mA g^{-1}, 100 cycles)	
NCM811+Li_6PS_5Cl (72:25)/Li_6PS_5Cl/Li-In	$LiZr_2(PO_4)_3$	Zr^{4+}	2.8–4.4 V	RT	185 (20 mA g^{-1})	99% (20 mA g^{-1}, 200 cycles)	[124]
NCM811+Li_6PS_5Cl (70:30)/Li_6PS_5Cl/Li-In	Li_7TaO_6	Ta	2.6–4.4 V	30 °C	131.25 (170 mA g^{-1})	61.1% (170 mA g^{-1}, 5650 cycles)	[125]
NCM811+Li_6PS_5Cl (70:30)/Li_6PS_5Cl/Li-In	Li_3PO_4	B^{3+}, P^{5+}	2.6–4.6 V	30 °C	121.75 (170 mA g^{-1})	76.8% (170 mA g^{-1}, 5650 cycles)	[93]
	Li_3PO_4	B^{3+}, P^{5+}	2.6–4.8 V	30 °C	133.64 (170 mA g^{-1})	53.28% (170 mA g^{-1}, 2000 cycles)	
	Li_3PO_4	B^{3+}, P^{5+}	2.6-4.8 V	-20 °C	52.1 (510 mA g^{-1})	90.27% (510 mA g^{-1}, 250 cycles)	
	Li_3PO_4	B^{3+}, P^{5+}	2.6–4.8 V	60 °C	82.7 (510 mA g^{-1})	51.03% (510 mA g^{-1}, 500 cycles)	
NCM811+Li_6PS_5Cl (70:30)/Li_6PS_5Cl/Li-In	Li_2ZrO_3	Al	2.7–4.4 V	30 °C	128.7 (360 mA g^{-1})	96.27% (360 mA g^{-1}, 1000 cycles)	[94]
Li_3InCl_6@NCM811+ Li_3InCl_6(60:36)/Li_6PS_5Cl/Li	Li_3InCl_6	Li_3InCl_6	2.5–4.2 V	RT	138 (20 mA g^{-1})	77.4% (40 mA g^{-1}, 100 cycles)	[107]

Table S4. Performance summary of NCM811–sulfide SSE-based ASSLB modified via sulfide SSE interface optimization.

Cathode/electrolyte /anode	Electrolyte	Method	Voltage range (V vs Li^+/Li)	Tempe -rature	Capacity (mAh g^{-1})	Cycle Performance	Ref.
NCM811+$Li_6PS_{4.7}O_{0.3}Br$ (50:50)/ $Li_6PS_{4.7}O_{0.3}Br$ /Li-In	$Li_6PS_{4.7}O_{0.3}Br$	O-doped Li_6PS_5Br	2.6–4.3 V	RT	108.7 (0.1 C)	77.4% (0.8 C, 100 cycles)	[77]
Li_3InCl_6@NCM811 + $Li_{20/3}(GeSiSb)_{1/3}S_5I$ (70:30)/ $Li_{20/3}(GeSiSb)_{1/3}S_5I$ /Li-In	$Li_{20/3}(GeSiSb)_{1/3}S_5I$	Ge, Si, and Sb substitution of P in Li_6PS_5I	2.6–4.3 V	25 °C	~219 (20 mA g^{-1})	~84.4% (200 mA g^{-1}, 550 cycles)	[95]
NCM811+Li_6PS_5Cl (60:35)/Li_6PS_5Cl/Li-In	Li_6PS_5Cl	Mechano-electrochemical healing	2.0–4.3 V	30 °C	~200 (20 mA g^{-1})	~95% (20 mA g^{-1}, 200 cycles)	[84]
NCM811+$Li_{5.7}PS_{4.7}Cl_{1.3}$ (70:30)/ $Li_{5.7}PS_{4.7}Cl_{1.3}$/Li-In	$Li_{5.7}PS_{4.7}Cl_{1.3}$	Cl-doped Li_6PS_5Cl	2.5–4.4 V	RT	140.8 (0.255 mA cm^{-2})	92.4% (0.255 mA cm^{-2}, 50 cycles)	[126]
NCM811+ $Li_6POS_4Br_{0.5}Cl_{0.5}$ (75:22)/ $Li_6POS_4Br_{0.5}Cl_{0.5}$/Li-In	$Li_6POS_4Br_{0.5}Cl_{0.5}$	O-doped $Li_6PS_5Br_{0.5}Cl_{0.5}$	2.0–3.7 V	55 °C	~200 (0.1 C)	~95.7% (0.3 C, 50 cycles)	[67]

References:

[116] X. Li, L. Jin, D. Song, H. Zhang, X. Shi, Z. Wang, L. Zhang, L. Zhu, *J. Energy Chem.* **2020**, *40*, 39.
[117] R. Tian, Z. Wang, J. Liao, H. Zhang, D. Song, L. Zhu, L. Zhang, *Adv. Energy Mater.* **2023**, *13*, 2300850.
[118] Y. Park, J. H. Chang, G. Oh, A. young Kim, H. Chang, M. Uenal, S. Nam, O. Kwon, *Small* **2024**, *20*, 2305758.
[119] X. Li, Z. Ren, M. Norouzi Banis, S. Deng, Y. Zhao, Q. Sun, C. Wang, X. Yang, W. Li, J. Liang, X. Li, Y. Sun, K. Adair, R. Li, Y. Hu, T. K. Sham, H. Huang, L. Zhang, S. Lu, J. Luo, X. Sun, *ACS Energy Lett.* **2019**, *4*, 2480.
[120] X. Li, J. Wang, C. Han, K. Zeng, Z. Wu, D. Wang, *Adv. Powder Mater.* **2024**, *3*, 100228.
[121] X. Liu, J. Shi, B. Zheng, Z. Chen, Y. Su, M. Zhang, C. Xie, M. Su, Y. Yang, *ACS Appl. Mater. Interfaces* **2021**, *13*, 41669.
[122] Y. Su, X. Liu, H. Yan, J. Zhao, Y. Cheng, Y. Luo, J. Gu, H. Zhong, A. Fu, K. Wang, M. sheng Wang, J. Huang, J. Yan, Y. Yang, *Nano Energy* **2023**, *113*, 108572.
[123] V. Mahajani, K. Bhimani, A. S. Lakhnot, R. Panchal, A. Anjan, R. M. Manoj, N. Koratkar, *Small* **2024**, *20*, 2402126.
[124] C. Zou, Z. Zang, X. Tao, L. Yi, X. Chen, X. Zhang, L. Yang, X. Liu, X. Wang, *ACS Appl. Energy Mater.* **2023**, *6*, 3599.
[125] C. Yu, Y. Li, M. Willans, Y. Zhao, K. R. Adair, F. Zhao, W. Li, S. Deng, J. Liang, M. N. Banis, R. Li, H. Huang, L. Zhang, R. Yang, S. Lu, Y. Huang, X. Sun, *Nano Energy* **2020**, *69*, 104396
